# Core-Clock Genes Regulate Proliferation and Invasion via a Reciprocal Interplay with MACC1 in Colorectal Cancer Cells

**DOI:** 10.3390/cancers14143458

**Published:** 2022-07-16

**Authors:** Alireza Basti, Deeksha Malhan, Malti Dumbani, Mathias Dahlmann, Ulrike Stein, Angela Relógio

**Affiliations:** 1 Charité—Universitätsmedizin Berlin, Corporate Member of Freie Universität Berlin and Humboldt—Universität zu Berlin, Institute for Theoretical Biology, 10115 Berlin, Germany; alireza.basti@charite.de (A.B.); deeksha.malhan@medicalschool-hamburg.de (D.M.); 2 Charité—Universitätsmedizin Berlin, Corporate Member of Freie Universität Berlin and Humboldt—Universität zu Berlin, Medical Department of Hematology, Oncology, and Tumor Immunology, Molecular Cancer Research Center, 13353 Berlin, Germany; 3 Institute for Systems Medicine, Faculty of Human Medicine, MSH Medical School Hamburg, 20457 Hamburg, Germany; 4 Translational Oncology of Solid Tumors, Experimental and Clinical Research Center, Charité-Universitätsmedizin Berlin and Max-Delbrück-Center for Molecular Medicine Berlin in the Helmholtz-Association, Robert-Rössle-Straße 10, 13125 Berlin, Germany; malti.dumbani@mdc-berlin.de (M.D.); dahlmann@mdc-berlin.de (M.D.); ustein@mdc-berlin.de (U.S.); 5 German Cancer Consortium, Im Neuenheimer Feld 280, 69120 Heidelberg, Germany

**Keywords:** circadian clock, colorectal cancer, invasion, EMT, core-clock manipulation

## Abstract

**Simple Summary:**

Colorectal cancer (CRC) belongs to the top three most common malignancies and is one of the deadliest cancers worldwide. Advancements in the understanding of CRC pathophysiology can lead to the development of novel treatments preventing cancer progression while prolonging overall survival. Numerous studies have shown a role for the biological clock in the regulation of cancer hallmarks and in CRC. However, the mechanistic link between the circadian clock and CRC progression is not fully understood. In the current study, we aimed to investigate the effects of a genetically disrupted clock on cancer properties using different CRC cell lines, with a focus on metastasis-related components. Our results demonstrate a reciprocal interplay between the circadian clock and the metastasis associated gene *MACC1* (metastasis-associated in colon cancer 1), pointing to the circadian clock-regulation of CRC invasiveness. A circadian MACC1 expression, as shown by our data, may be considered to optimize MACC1-targeted CRC treatment.

**Abstract:**

The circadian clock coordinates the timing of several cellular processes including transcription, the cell cycle, and metabolism. Disruptions in the clock machinery trigger the abnormal regulation of cancer hallmarks, impair cellular homeostasis, and stimulate tumourigenesis. Here we investigated the role of a disrupted clock by knocking out or knocking down the core-clock (CC) genes *ARNTL*, *PER2* or *NR1D1* in cancer progression (e.g., cell proliferation and invasion) using colorectal cancer (CRC) cell lines HCT116, SW480 and SW620, from different progression stages with distinct clock phenotypes, and identified mechanistic links from the clock to altered cancer-promoting cellular properties. We identified *MACC1* (metastasis-associated in colon cancer 1), a known driver for metastasis and an EMT (epithelial-to-mesenchymal transition)-related gene, to be significantly differentially expressed in CC manipulated cells and analysed the effect of *MACC1* manipulation (knockout or overexpression) in terms of circadian clock phenotype as well as cancer progression. Our data points to a bi-directional *MACC1*-circadian clock interplay in CRC, via CC genes. In particular, knocking out *MACC1* reduced the period of oscillations, while its overexpression increased it. Interestingly, we found the MACC1 protein to be circadian expressed in HCT116 WT cells, which was disrupted after the knockout of CC genes, and identified a MACC1-NR1D1 protein–protein interaction. In addition, *MACC1* manipulation and CC knockout altered cell invasion properties of HCT116 cells, pointing to a regulation of clock and cancer progression in CRC, possibly via the interaction of *MACC1* with core-clock genes.

## 1. Introduction

To further develop and form metastasis in the organism, cancer cells must escape a series of safeguard mechanisms leading to a failure in several key cellular functions, from apoptosis to DNA damage response, which are known as the hallmarks of cancer [1]. Recent studies have shown that several, if not all, of these hallmarks are under circadian control [2]. In mammals, up to 80% of protein-encoding genes are clock-controlled and expressed in a circadian manner in at least one tissue [3,4]. As a result, clock disruption affects cellular homeostasis and may predispose individuals to cancer [5,6,7].

On the molecular level, the circadian clock generates and maintains robust rhythmic expression in genes and proteins via interlocking transcriptional/translational feedback loops of core-clock (CC) genes including *CLOCK*, *ARNTL* (aka *BMAL1*), *PER1/2*, *CRY1/2* and *NR1D1/2*, which subsequently regulate the circadian expression of the so-called clock-controlled genes (CCGs) [5]. These genes include well-known oncogenes and tumour suppressors involved in the cell cycle (e.g., *MYC*) [8], cell death (e.g., *TP53*) [9] and cell growth (e.g., *RAS*) [10,11].

In cancer, several CC genes, including *CLOCK*, *ARNTL*, *PER2* and *NR1D1* are dysregulated and play a role in tumourigenesis (reviewed in [12]). In colorectal cancer (CRC) in particular, the expression of CC genes is altered in patient samples [13], as well as in cancer cell lines [10,14]. While *ARNTL*, *PER1* and *CRY2* seem to have oncogenic effects in human CRC cells [14,15,16], *CRY1* is thought to be tumour-suppressive upon silencing [17]. These findings point to a dysregulation of the circadian clock in CRC, with different effects upon alterations in CC genes.

The clinical importance of these findings has been demonstrated in several studies, which show a prominent role for circadian-based therapy in CRC patients, with increased efficacy and survival rate compared with conventional therapy [18,19]. Additionally, circadian rest-activity cycles measured through actigraphy and wrist accelerometers have been used as biomarkers to monitor and predict treatment responses and patient outcomes in colorectal cancer [20,21].

In our previous work, we investigated the role of circadian clock components in altering the expression of genes related to cancer hallmarks in CRC cell lines and pointed to a role for *ARNTL* and *NR1D1* in regulating cancer growth and apoptosis, as well as metastasis potential [22]. This led us to the hypothesis that the circadian clock regulates genes related to cancer metastasis via several pathways, including epithelial-to-mesenchymal transition (EMT), cell proliferation and cell invasion. Among the several related genes, we focused on metastasis-associated in colon cancer 1 (*MACC1*), known to be a driver for cancer metastasis, especially in CRC [23,24], and for which no connection to the circadian clock has been established to our knowledge.

In the present work, we sought to investigate the extent of circadian clock control in CRC cell progression (e.g., proliferation and migration) via CC genes, as well as CCGs, which regulate cell migration and invasiveness. We used CRC cell lines of different progression stages and origins with distinct clock phenotypes (HCT116 and SW480 from primary tumour as well as SW620, the metastatic counterpart of SW480). Bioluminescence live-cell measurements show that HCT116 cells display a robust circadian oscillation compared with SW480 and SW620 cell lines that have a moderate and weak oscillation pattern, respectively [10,22,25], providing an interesting in vitro model for further investigating the effect of CC perturbations using different CRC oscillators.

We generated CC manipulated CRC cells (*ARNTL*, *PER2* or *NR1D1* knockout or knockdown) and compared clock (*ARNTL*-promoter activity) and cancer phenotype (proliferation, apoptosis and invasion) to that of *MACC1* manipulated (knockout or overexpression) cells. The CC manipulations led to differential expression of several key EMT genes, among them *MACC1*, and affected cancer proliferation and migration. Interestingly, we found that *MACC1* alters the circadian phenotype and modulates the cell intrinsic period in CRC. We further detected a circadian oscillation in MACC1 protein expression, which was lost upon CC knockout in HCT116, as well as a protein–protein interaction between MACC1 and NR1D1. Hence, we provide evidence of a reciprocal interplay between *MACC1* and circadian clock using our in vitro cellular model of CRC, with an impact on CRC progression, in particular cancer cell proliferation and invasion.

## 2. Materials and Methods

### 2.1. Cell Culture

HCT116 (ATCC^®^ CCL-247™), SW480 (ATCC^®^ CCL-228™) and SW620 (ATCC^®^ CCL-227™) cells were cultured in Dulbecco’s Modified Eagle Medium DMEM (Gibco, Thermo Fisher Scientific, Waltham, MA, USA) supplemented with 10% FBS (Gibco, Thermo Fisher Scientific, Waltham, MA, USA) and 1% Penicillin−Streptomycin (Gibco, Thermo Fisher Scientific, Waltham, MA, USA) in a humidified atmosphere containing 5% CO_2_ at 37 °C. *MACC1* overexpressing SW480 and HCT116 cells were generated as previously described by [23] and [26], respectively. The generation of *MACC1* knockout in SW620 cells was performed as previously described [27].

### 2.2. CRISPR-Cas9 Knockout Generation in HCT116

To generate core-clock knockout cells in HCT116, CRISPR-Cas9 methodology was applied. Briefly, HCT116 WT cells were seeded in 6-well plates with a density of 4 × 10^5^ cells/well and transfected with CRISPR-Cas9 plasmids containing a GFP tag and guided RNAs (gRNAs) targeting multiple exons of *ARNTL*, *PER2* or *NR1D1* genes, respectively. A list of gRNA sequences and Cas9 plasmid types can be found in Table 1. For cell transfection, FuGENE HD Transfection Reagent (Promega Corporation, Fitchburg, WI, USA) was used, according to the manufacturer’s instructions. GFP-positive cells were single-cell sorted into 96-well plates 48 h post transfection using an S3e cell sorter (Bio-Rad laboratories, Hercules, CA, USA), expanded and evaluated for knockout success on DNA, RNA and protein levels.

CRISPR-Cas9 off-target activity was evaluated using Off-Spotter [28] and Welcome Trust Sanger Institute Genome Editing database (WGE) [29] online tools, to search for the most likely potential off-target sites based on gRNA sequences. We searched for off-target sites with up to three mismatches and within protein-coding regions, Sanger-sequenced them and compared the sequence to WT. All investigated potential off-target sites in knockout cells showed 100% sequence similarity to WT, indicating no off-target modifications (Table 2). 

Stable transduced cells were selected and maintained in medium containing 150 μg/mL hygromycin B (Gibco, Thermo Fisher Scientific, Waltham, MA, USA) for the *ARNTL*:LUC hygromycin and 1.5 μg/mL of puromycin (Gibco, Thermo Fisher Scientific, Waltham, MA, USA) for *ARNTL*:LUC puromycin as well as the shRNA KD of the clock genes. For live-cell bioluminescence recording, cells were maintained in phenol red-free DMEM (Gibco, Thermo Fisher Scientific, Waltham, MA, USA) containing 10% FBS, 1% Penicillin−Streptomycin and 250µM D-Luciferin (Bio-Rad laboratories, Hercules, CA, USA). Cell counting and morphology analysis were performed in a LUNA™ Automated Cell Counter (Logos Biosystems, Anyang, Korea). Cell lines were tested for mycoplasma using the Mycoplasmacheck service of Eurofins Genomics (Eurofins Genomics, Ebersberg, Germany).

### 2.3. Lentivirus Production

Lentiviral elements containing a *ARNTL*-promoter-driven luciferase, an empty vector (TRC Lentiviral pLKO.1 Empty Vector Control; Dharmacon Inc., Lafayette, CO, USA) or shRNA KD (TRC Lentiviral Human ARNTL shRNA—Clone ID: TRCN0000019096/97; TRC Lentiviral Human PER2 shRNA—Clone ID: TRCN0000018542; TRC Lentiviral Human NR1D1 shRNA—Clone ID: TRCN0000022174; Dharmacon Inc., CO, USA) were used in this work. For lentivirus production, HEK293T (human, kidney, ATCC Number: CRL-11268) cells were seeded in 175 cm culture flasks and co-transfected with 12.5 μg packaging plasmid psPAX, 7.5 μg envelope plasmid pMD2G and 17.5 μg expression plasmid using the CalPhos mammalian transfection kit (Clontech, Mountain View, CA, USA) according to the manufacturer’s instruction. To harvest the lentiviral particles, the supernatant was centrifuged at 4100× *g* for 15 min to remove cell debris and passed through a 45 μm filter (Sarstedt, Nümbrecht, Germany). The lentiviral particles were stored at −80 °C.

### 2.4. Transduction with Lentiviral Vectors

For lentiviral transduction, 1 × 10^5^ cells were seeded in 6-well plates. On the day of transduction, 1.5 mL of supernatant of the corresponding lentivirus was added to each well. We used 8 μg/mL protamine sulfate (Sigma-Aldrich, St. Louis, MO, USA) and 4 μg/mL polybrene (Sigma-Aldrich, St. Louis, MO, USA) to enhance transduction efficiency. After 48 h, the medium was replaced and the selection medium was added (complete growth medium containing appropriate antibiotic) to obtain stably transduced cells and incubated at 37 °C with 5% CO_2_ atmosphere. Untransduced cells treated with the same antibiotic concentration were used as selection controls.

### 2.5. Bioluminescence Measurements

For live-cell bioluminescence recordings, 2.5 × 10^5^ cells were seeded in 35 mm dishes and maintained in phenol red-free DMEM (Gibco, Thermo Fisher Scientific, Waltham, MA, USA) containing 10% FBS, 1% Penicillin–Streptomycin supplemented with 250 µM D-Luciferin (Bio-Rad laboratories, Hercules, CA, USA). Cells were synchronized by medium change prior to measurement (zeitgeber time = 0 h). *ARNTL*-promoter-reporter activities were measured using a LumiCycle instrument (Actimetrics, Wilmette, IL, USA) for five consecutive days. Raw luminescence data were de-trended by the 24 h running average (divided values) using the Chronostar analysis software V3.0 [30]. The first 12 h of measurement were removed from the analysis, since the first data collection is comparatively very noisy due to technical limitations of the device. The phase in radian was calculated using the following equation:

φ(rad) = φ(h)⋅(2⋅π/T)(1)

where φ(h) = phase (in h) and T = period.

### 2.6. RNA Extraction, cDNA Synthesis (Reverse Transcription) and Quantitative Real-Time PCR (qPCR)

Total RNA was isolated using the RNeasy Plus Mini kit (Qiagen, Hilden, Germany) according to the manufacturer’s manual. Prior to the purification procedure, the medium was discarded and the cells were washed with PBS and lysed in RLT Plus buffer (Qiagen, Hilden, Germany). The genomic DNA was digested using gDNA eliminator columns provided with the kit (Qiagen, Hilden, Germany). RNA was eluted in 25–50 µL RNase-free water. The final RNA concentration was measured using a Nanodrop 1000 (Thermo Fisher Scientific, Waltham, MA, USA). The RNA was then stored at −80 °C until use. Next, 1 µg of total RNA was reverse-transcribed to cDNA with M-MLV reverse transcriptase (Invitrogen, Thermo Fisher Scientific, Carlsbad, CA, USA), random hexamers (Thermo Fisher Scientific, Waltham, MA, USA) and dNTPs Mix (Thermo Fisher Scientific, Waltham, MA, USA). RT-qPCR was performed using human QuantiTect Primer assays (Qiagen, Hilden, Germany), unless otherwise indicated (see primer list in Table 3), and SsoAdvanced Universal SYBR Green Supermix (Bio-Rad laboratories, Hercules, CA, USA) in 96-well plates. GAPDH was used as reference gene. The qPCR reaction and the subsequent melting curve were performed using a CFX Connect Real-Time PCR Detection System (Bio-Rad laboratories, Hercules, CA, USA). A melting curve analysis was performed to detect potential unspecific amplification products. Cq values were determined using the regression method. The expression levels were normalised to those of *GAPDH* (ΔCT) and calibrated to the mean expression value of each gene (time-course analysis) or in relation to the respective control (ΔΔCT). Relative quantification was calculated using the 2^−ΔΔCt^ method. Biological and technical replicates were included in the analysis. The mean and the standard error of the mean were calculated.

### 2.7. Western Blotting and Immunoprecipitation

Cells were synchronized by medium change, gently detached from the dish, sedimented by low-speed centrifugation and resuspended in lysis buffer containing Halt Protease and Phosphatase inhibitors (1×, Thermo Fisher Scientific, Waltham, MA, USA). Aliquots containing 30 µg of proteins from each cell lysate were subjected to SDS polyacrylamide gel electrophoresis and transferred to a Nitrocellulose Membranes (Bio-Rad laboratories, Hercules, CA, USA) using the Trans-Blot Turbo Transfer System (Bio-Rad laboratories, Hercules, CA, USA). The membranes were probed with the following primary antibodies: ARNTL (1/2000, ab93806, Abcam, Cambridge, UK); PER2 (1/250, LS-C358004, LSBio, Seattle, WA, USA); NR1D1 (1/1000, ab174309, Abcam); MACC1 (1/10000, HPA020081, Sigma-Aldrich, St. Louis, MO, USA); and GAPDH (1/2500; ab9485, Abcam). After incubation with the corresponding secondary antibody (1/2000; ab205718, Abcam), signals were detected using the Amersham ECL Select Western Blotting Detection Reagent (GE Health care, Chicago, IL, USA), acquired by Image Quant LAS 4000 series (GE Health care). The data was analysed using imageJ v1.48 [31].

For the protein–protein interaction assay, Dynabeads™ Protein G beads were used according to the manufacturer’s instructions (Invitrogen). In short, 1500 μg total protein was incubated with the NR1D1 antibody (2 μg, #13418, Cell Signaling, Danvers, MA, USA), the ARNTL antibody (2 µg ab93806, Abcam, Cambridge, UK) or an isotype control (ab172730, Abcam) overnight and pulled down using protein G magnetic beads. After elution, MACC1 (1/5000, HPA020081, Sigma-Aldrich, St. Louis, MO, USA) was detected by Western blotting.

### 2.8. Cell Cycle Assay

Synchronized cells under logarithmic growth phase were collected 24 h post synchronization, washed once with PBS and fixed using ice cold 100% ethanol in PBS. The samples were kept at −20 °C for at least 24 h. The fixed cells were washed twice with cold PBS and incubated in 200 µL PBS in the presence of RNase (0.25 mg/mL, Thermo Scientific, Waltham, MA, USA) for 30 min at 37 °C. For DNA staining, the cells were washed once with PBS and stained with 500 µL PI solution (50 µM, Invitrogen, Waltham MA, USA) in PBS for 30 min at 37 °C. Subsequently, the supernatant containing the PI solution was removed and the stained cells were resuspended in 500 µL PBS and read in BD FACSCanto™ II (Becton Dickinson, Franklin Lakes, NJ, USA). The cell cycle analysis was conducted by fitting a univariate cell cycle model using the Watson pragmatic algorithm as implemented in FlowJo v10.8 (FlowJo LLC, Ashland, OR, USA). It should be noted that the cell cycle assay provides a snapshot of cell percentages in different phases and gives valuable biological insights into cell dynamics, which are not directly comparable to a high-resolution live-cell proliferation assay.

### 2.9. Proliferation Assay

For the proliferation assay, 5000 cells/well were seeded in a 96-well plate (Sarstedt, Nümbrecht, Germany), with cells having similar confluence at T0 of the experiment. This allowed for a comparison of growth rate over time using cell confluence. The cells were allowed to adhere and placed in the IncuCyte^®^ S3 Live Cell System Analysis (Sartorius, Göttingen, Germany). Four pictures were recorded every two hours for biological and technical replicates. The analyses were performed by using IncuCyte^®^ S3 Software (Sartorius, Göttingen, Germany). We also calculated the cell doubling time for the HCT116 CC KO cells: WT (23.24 h ± 0.12), *ARNTL* KO (22.3 h ± 00.13), *PER2* KO (20.76 h ± 0.19) and *NR1D1* KO (23.20 h ± 0.14). Cell duplication time was calculated using the following formula:

$$\mathrm{doubling}\;\mathrm{time}=\mathrm{duration}\;\times \frac{\ln\left(2\right)}{\ln\left(\mathrm{final}\;\mathrm{confluency}\right)/\ln\left(\mathrm{initial}\;\mathrm{confluency}\right)}$$

### 2.10. Apoptosis Assay

Cells were seeded in a 96-well plate (Sarstedt, Nümbrecht, Germany) at a concentration of 5000 cells/100 µL medium and incubated for 24 h in an incubator at 37 °C with 5% CO_2_. For each cell line, biological replicates and technical replicates were prepared. After 24 h incubation, the cell media were replaced with fresh medium containing caspase 3/7 (Sartorius, Göttingen, Germany, 1/2000). Cell apoptosis was measured using the IncuCyte^®^ S3 Live Cell System Analysis. The cells were scanned every 3 h with a 10× objective using the phase and fluorescent green image channels.

### 2.11. Migration Assay

For the migration assay, 35,000 cells/well were seeded in a 96-well Essen ImageLock^TM^ microplate (Sartorius, Göttingen, Germany) and incubated overnight at 37 °C, 5% CO_2_. The following day, the WoundMaker^TM^ (Sartorius, Göttingen, Germany) was used to create precise and reproducible wounds. Image acquisition was performed by setting the “scan type” to Scratch Wound and Wide Mode, using the 10× objective. The plate was scanned every two hours. Analysis was performed with the scratch wound method in the IncuCyte S3^®^ Software (Sartorius, Göttingen, Germany) and by measuring the relative wound density over time. Relative wound density measures the percentage of spatial cell density in the wound area relative to the spatial cell density outside of the wound area at each time point, allowing normalization for changes in cell density caused by cell division and is measured as following:

$$\%\mathrm{RWD}\left(t\right)=100\times \frac{\left(w\left(t\right)-w\left(0\right)\right)}{\left(c\left(t\right)-w\left(0\right)\right)}$$

where w(t) is the density of the wound region at time t, and c(t) is the density of the cell region at time t.

### 2.12. Chemotaxis Invasion Assay

To evaluate cell invasion potential, the IncuCyte^®^ Chemotaxis Cell Invasion Assay, which evaluates chemotactic cell invasion through a biomatrix, was used according to the manufacturer’s instructions. For this, cells were harvested and mixed with the assay medium (1% FBS) containing reduced growth factor Basement Membrane Extract (Trevigen, MD, USA) with a final concentration of 5 mg/mL and seeded into the insert of a primed 96-well IncuCyte^®^ Clearview Plate (Sartorius, Göttingen, Germany) with 2000 cells/well. The Clearview Plate was centrifuged and incubated at 37 °C for 60 min to polymerize the biomatrix. Finally, the insert was transferred into a preloaded reservoir plate containing 200 µL complete medium (10% FBS). The plate was placed in an IncuCyte S3^®^ device and scanned using the Chemotaxis scan type (imaging the top and bottom layer of the insert) every 2 h with a 10× objective.

### 2.13. Rhythmicity Analysis

Circadian rhythms and circadian related parameters (amplitude, acrophase) in protein data were determined using the Cosinor analysis within the Discorhythm R package (version 1.10.0 [32]). Statistical significance for 24 h rhythmic protein was set at *p* ≤ 0.05. It should be noted that the plotted data are GAPDH and mean normalized in order to minimize the influence of technical effects and detect biological circadian oscillations within the time-series interval.

### 2.14. Differential Correlation Analysis

A Pearson correlation was calculated for the set of core clock genes and EMT genes in different cell line datasets using the R package “corrr()”. To further understand the differences in the correlation between gene pairs across multiple conditions, a differential correlation analysis was carried out using “DGCA” (differential gene correlation analysis) R package (version 1.0.2 [33]). The function “DiffCorr()” was used to calculate correlations in each condition using z-transformed correlation coefficients to calculate *p*-values (two-tailed *t*-test).

### 2.15. Statistical Analysis

Experiments were carried out with at least three biological replicates for each condition. All the data is presented as mean ± SEM. * *p* < 0.05, ** *p* < 0.01, *** *p* < 0.001. The statistical analysis was performed using Prism software (GraphPad version 8, GraphPad Software, San Diego, CA, USA). Proliferation, migration and apoptosis was analysed by comparing the area under the curve (AUC) between the control and manipulated conditions and tested using a two-tailed unpaired *t*-test.

## 3. Results

### 3.1. Core-Clock Manipulation Disrupts the Circadian Clock Network and Affects Expression of Genes Involved in Cell Cycle, EMT and Migration

**Figure 1:**
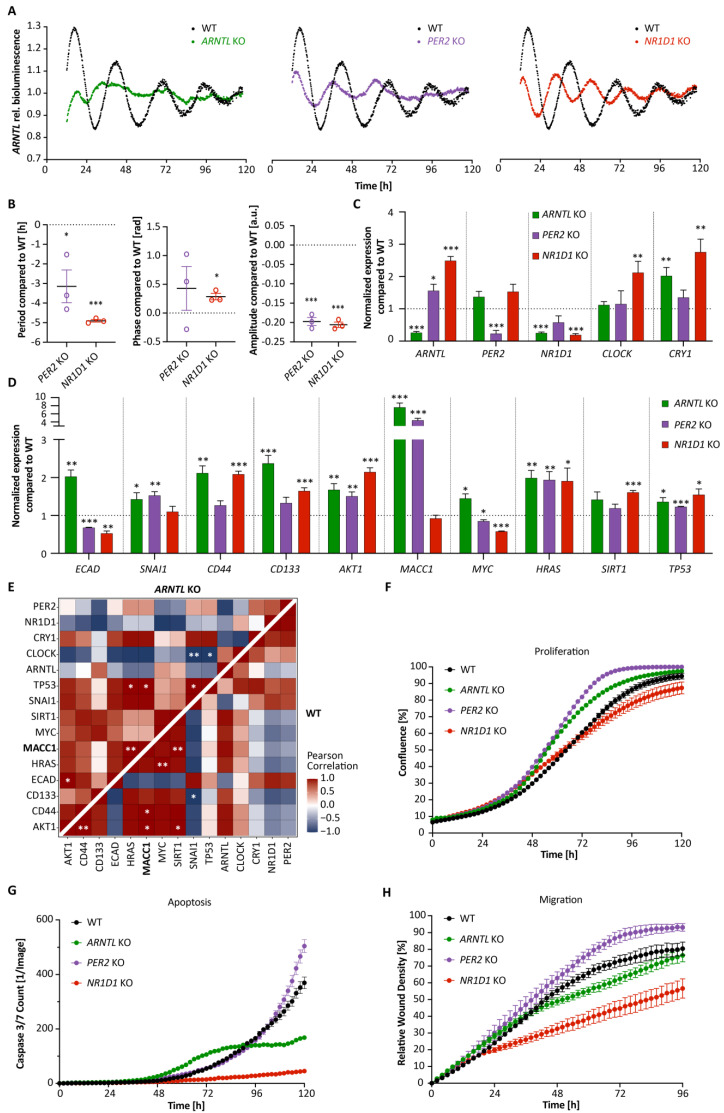
*ARNTL* promoter activity shows different oscillation patterns in HCT116 knockout cell lines. (**A**) Bioluminescence readouts for the promoter activity of *ARNTL* over the course of 120 h in HCT116 WT and CC knockout (*ARNTL* KO, *PER2* KO and *NR1D1* KO) cell lines. (**B**) Period, phase and amplitude analysis of bioluminescence data of HCT116 knockout cells using Chronostar (*n* = 3, mean ± SEM). T_WT_ = 26.1 ± 0.1 h, T*_ARNTL_* _KO_ = ND, T*_PER2_*_KO_ = 23.0 ± 0.8 h, T*_NR1D1_*_KO_ = 21.2 ± 0.1 h. (**C**) Gene expression analysis of CC genes *PER2*, *CRY1*, *NR1D1*, *CLOCK*, and *ARNTL* in HCT116 WT and knockout cell lines at 24 h after synchronization (*n* = 3, mean ± SEM). (**D**) Gene expression analysis of related genes in EMT, cell cycle, death and metastasis in HCT116 CC knockout cell lines at 24 h after synchronization (*n* = 3, mean ± SEM). (**E**) Heatmaps of Pearson correlation between each pair of CC and EMT-related genes for HCT116 WT versus *ARNTL* KO. (**F**–**H**): Proliferation (**F**), Apoptosis (**G**) and Migration (**H**) analysis of HCT116 WT and CC knockouts using live-cell imaging over several days (*n* > 8, mean ± SEM, significance tested by comparing AUC to WT, two-tailed unpaired *t*-test). ND: not defined. * *p* < 0.05, ** *p* < 0.01, *** *p* < 0.001, two-tailed unpaired *t*-test.

**Figure 2:**
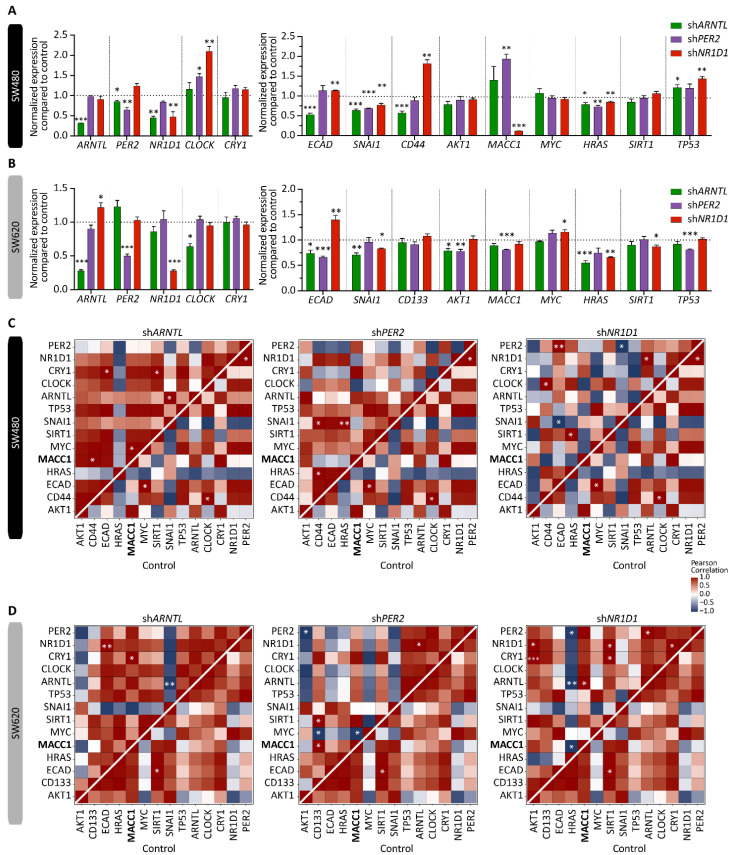
Core-clock manipulation affects EMT gene expression in SW480 and SW620 cells. Gene expression analysis of core-clock genes *PER2*, *CRY1*, *NR1D1*, *CLOCK*, and *ARNTL* as well as EMT-related genes in control and core-clock knockdown (sh*ARNTL*, sh*PER2* and sh*NR1D1*, respectively) cell lines in SW480 (**A**) and SW620 (**B**) cells at 24 h after synchronization (*n* = 3, mean ± SEM). Heatmaps of Pearson correlations between each pair of core-clock and EMT-related genes for (**C**) SW480 and (**D**) SW620 sh*ARNTL*, sh*PER2* and sh*NR1D1* cells compared with the control cell line. * *p* < 0.05, ** *p* < 0.01, *** *p* < 0.001; two-tailed unpaired *t*-test.

To investigate the putative effect of a disrupted circadian clock machinery in cancer-associated properties in human CRC cells, in particular EMT and cell migration, we established knockout (KO) mutants for *ARNTL*, *PER2* or *NR1D1* using CRISPR-Cas9 in HCT116 cells, as well as stable knockdowns (KD) of the same genes in SW480 and SW620 cell lines, and investigated both clock- and cancer cell-related phenotypes (Figure 1 and Figure 2; Supplementary Figure S1 and S2). KO of *ARNTL* abolished *ARNTL*-promoter activity, and *PER2* KO and *NR1D1* KO significantly reduced the period of the oscillations in HCT116, in agreement with previous findings [34,35] (Figure 1B, ∆T*_PER2_*_KO_ = −3.1 ± 0.8 h and ∆T*_NR1D1_* _KO_ = −4.9 ± 0.1 h, mean ± SEM, *n* = 3, *p* < 0.05). As reported in previous studies, SW480 cells display a moderate oscillation pattern and SW620 cells are considered as weak oscillators [25,36,37]. The CC gene KO resulted in differential expression of other CC genes (including *CLOCK* and *CRY1*), as well as several EMT-related genes (Figure 1C,D). In particular, *ARNTL* KO reduced *NR1D1* (*p* < 0.001) and increased *CRY1* (*p* < 0.01) expression significantly, as previously reported [22], and *NR1D1* depletion resulted in significant upregulation in *ARNTL* (*p* < 0.001), *CLOCK* and *CRY1* (*p* < 0.01). We further investigated the effect of CC KO in elements of cancer- and metastasis-related pathways such as the cell cycle (*MYC*), cell proliferation (*HRAS*), cell death (*TP53*), EMT (*ECAD* and *SNAI1*), metastasis (*CD44*, *CD133* and *MACC1*), as well as clock- and cancer-related genes (*SIRT1* and *AKT1*). All genes were significantly differentially expressed in at least one of the CC KOs. In particular, *MACC1* showed the strongest difference in HCT116 cells, with a more than 3-fold increase in *ARNTL* KO and *PER2* KO, and a slight downregulation after *NR1D1* KO in HCT116 (Figure 1D). We also observed significant differential expression of *MACC1* in SW480 and SW620 cells upon the stable downregulation of CC genes (Figure 2A,B). While *MACC1* was significantly upregulated in SW480 sh*PER2* cells (*p* < 0.01), it was significantly reduced in SW480 sh*NR1D1* and SW620 sh*PER2* cells (*p* < 0.001).

In addition, we quantified the extent of circadian perturbation in the CRC cell lines by computing the Pearson correlation between the expression values of each pair of CC and EMT-related genes and comparing it to the WT cells (Figure 1E and Figure 2C,D; Supplementary Figure S2).

In HCT116 WT, one set of genes (*AKT1*, *CD44*, *CD133*, *HRAS*, *MACC1*, *MYC*, *SIRT1*, and *ARNTL*) showed positive correlation within the group, and negative correlation with other set of genes (*ECAD*, *SNAI1*, *CRY1*, *NR1D1*, and *PER2*). Moreover, we also observed significant correlation between certain gene pairs such as *AKT1*-*MACC1* (*p* < 0.05) and *SIRT1*-*MACC1* (*p* < 0.01) (Figure 1E). All CC KOs resulted in changes in correlation patterns vs. WT. For instance, *ARNTL* KO resulted in loss of correlation between *CD133-MACC1* and *CD133*-*HRAS* (Figure 1E).

A set of genes (*AKT1*, *CD44*, *HRAS*, *MACC1*, *MYC*, *SIRT1*, *TP53*) showed positive correlation with the *CLOCK* gene in WT, whereas they were negatively correlated with the *CLOCK* gene in *ARNTL* KO. Similarly, the KO of other CC elements, *PER2* and *NR1D1* in HCT116 cells also resulted in a discrepant pattern of correlation vs. WT (Supplementary Figure S2). For instance, *PER2* KO showed a negative correlation between the *AKT1* gene and gene set (*PER2*, *NR1D1*, *CLOCK*, *ARNTL*, *SNAI1*, *SIRT1*, *MACC1*, *CD133*, and *CD44*), whereas *NR1D1* KO showed a positive correlation in the same gene-pairs, pointing towards the KO-specific changes in gene correlation patterns. We also found *AKT1-ARNTL* positively correlated in HCT116 WT and SW480 control cells, whereas it was negatively correlated in *ARNTL* KO and *PER2* KO in HCT116 and SW620 control cells, which might point to an alteration of clock regulation related to cancer progression.

Interestingly, *MACC1* correlation changed under different KO conditions vs. WT. For instance, whereas *MACC1* showed a strong positive correlation with *ARNTL* and negative correlations with *NR1D1*, *PER2* and *ECAD* in WT, we observed the opposite pattern after *ARNTL* KO (Figure 1E).

The different KOs also resulted in alterations in proliferation, apoptosis and migration capability of the HCT116 cells (Figure 1F–H). Notably, proliferation was increased upon *ARNTL* KO and *PER2* KO (*p* < 0.001, AUC compared with WT) and was not significantly altered after *NR1D1* KO (Figure 1F). Interestingly, *ARNTL* KO and *NR1D1* KO both significantly reduced cell apoptosis and migration (*p* < 0.001, AUC compared to WT) which together with dysregulated expression of EMT related genes points towards the regulation of CC and *MACC1* in CRC possibly affecting cell motility (Figure 1G,H). It should be noted that the observed increase in cell apoptosis for *PER2* KO cells after 96 h is mainly due to cells reaching full confluence, as seen from cell proliferation data (Figure 1F). Taken together, our results show a KO-specific role of CC genes in regulating cancer phenotype and affecting both EMT gene expression and correlation patterns, and highlight *MACC1* as being strongly impacted, especially via *ARNTL* KO.

### 3.2. MACC1 Affects Both the Cellular Circadian Clock and Cancer Properties

**Figure 3:**
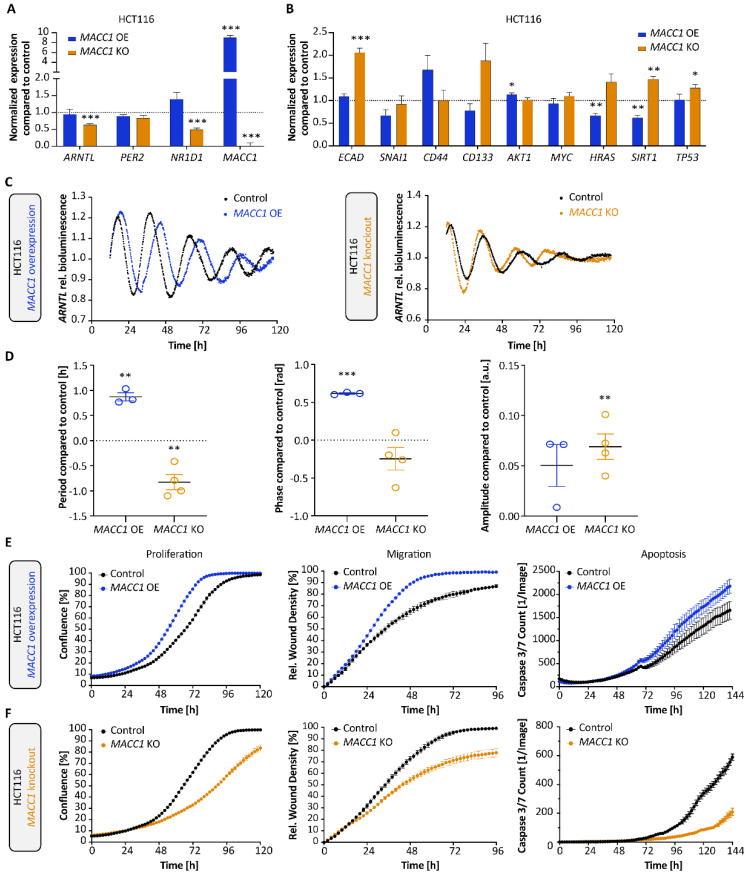
*MACC1* manipulation affects clock phenotype in HCT116 cells. Gene expression analysis of CC genes *ARNTL*, *PER2* and *NR1D1* (**A**) as well as EMT-related genes (**B**) in HCT116 *MACC1* overexpressing (OE) and *MACC1* knockout (KO) cells, respectively. Samples were collected at 24 h after synchronization (*n* = 3, mean ± SEM). (**C**,**D**) *ARNTL* promoter activity in HCT116 *MACC1* OE and HCT116 *MACC1* KO cell lines. Measurements were taken over five days using live-cell bioluminescence recordings. Period, phase and amplitude were measured using Chronostar (*n* ≥ 3, mean ± SEM). T_Control_ = 24.9 ± 0.1 h and T*_MACC1_*_OE_ = 25.8 ± 0.1 h, T_Control_ = 23.5 ± 0.1 h and T*_MACC1_*_KO_ = 22.4 ± 0.2 h. * *p* < 0.05, ** *p* < 0.01, *** *p* < 0.001; two-tailed unpaired *t*-test. (**E**,**F**) Proliferation, migration and apoptosis analysis of HCT116 *MACC1* OE (**E**) and HCT116 *MACC1* KO (**F**) cell lines using live-cell imaging (*n* > 8, mean ± SEM, significance tested by comparing AUC with the respective control cell line, two-tailed unpaired *t*-test).

**Figure 4:**
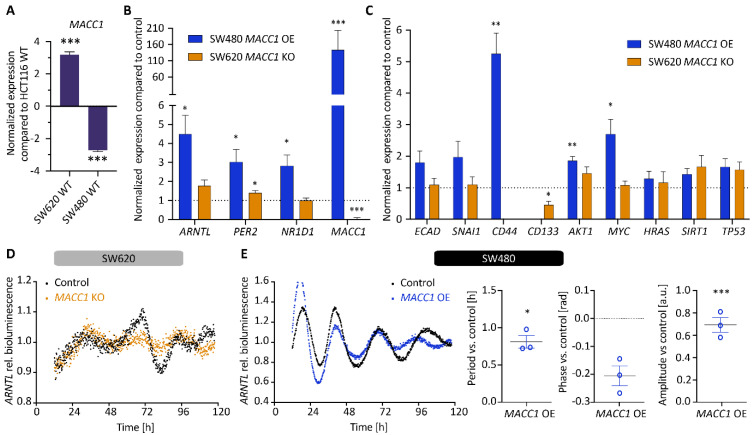
*MACC1* manipulation in SW480 and SW620 cell lines leads to differential expression in clock and EMT-related genes (**A**) Analysis of *MACC1* expression between HCT116 WT, SW480 WT and SW620 WT (*n* = 3, mean ± SEM). Gene expression analysis of core-clock genes *ARNTL*, *PER2* and *NR1D1* (**B**) as well as related genes in EMT, cell cycle, death and metastasis (**C**) in SW480 *MACC1* overexpressing (OE) and SW620 *MACC1* knockout (KO) cells, respectively. Samples were collected at 24 h after synchronization. Each condition was compared with its respective control cell line. (*n* = 3, mean ± SEM). (**D**,**E**) *ARNTL*-promoter activity in SW620 *MACC1* knockout (**D**) and SW480 *MACC1* overexpressing (**E**) cell lines compared with the respective control condition. T_control_ = 24.2 ± 0.3 h and T*_MACC1_*_OE_ = 25.0 ± 0.1 h, mean ± SEM. Measurements were made over five days using live-cell bioluminescence readouts. Period, phase and amplitude were measured using Chronostar. (*n* = 3, mean ± SEM). * *p* < 0.05, ** *p* < 0.01, *** *p* < 0.001; two-tailed unpaired *t*-test.

As described above, genes involved in EMT and metastasis pathways, in particular *MACC1,* are dysregulated upon manipulation of the CC genes *ARNTL*, *PER2* or *NR1D1*. To further assess the potential role of the CC on cancer metastasis, we focused on *MACC1* that showed the most striking expression change upon the KOs. For this, we analysed a cellular model of HCT116 *MACC1* overexpression (OE) and KO cells (Figure 3), as well *MACC1* manipulated SW480 and SW620 cells (*MACC1* OE and KO, respectively), as depicted in Figure 4. Our gene expression analysis points to a putative correlation between *ARNTL*, *NR1D1* and *MACC1* expression (Figure 3A and Figure 4B). While *MACC1* KO reduced *NR1D1* and *ARNTL* expression in HCT116 (*p* < 0.001), its overexpression significantly increased *ARNTL* and *NR1D1* in SW480 (*p* < 0.05), together with a slight induction of *NR1D1* in HCT116. Additionally, we observed a negative correlation between *MACC1* and two cancer and clock modulators, namely *HRAS* and *SIRT1*, known to affect the period of oscillation [10,38,39]. While *MACC1* OE reduced the expression of *HRAS* and *SIRT1*, its depletion increased their expression (Figure 3B). Of note is that endogenous *MACC1* expression was different among the three CRC WT cell lines, with SW620 WT cells expressing the most, followed by HCT116 WT and SW480 WT cells (Figure 4A).

We also observed differences among gene pairs in terms of their correlation coefficients in *MACC1* KO and *MACC1* OE conditions (Supplementary Figure S3). In HCT116, *MACC1* KO led to a negative correlation in only two gene pairs (*TP53*-*PER2* and *MYC*-*PER2*), whereas its OE in HCT116 cells showed positive correlation among the same two gene pairs. In addition, *MACC1* OE showed a general negative correlation pattern between all gene-pairs, in contrast to *MACC1* KO (Supplementary Figure S3A). When focusing on CC gene expression, *MACC1* KO resulted in a significant positive correlation in *NR1D1-ARNTL* (*p* < 0.01), which was lost in *MACC1* OE.

*ECAD* showed negative correlation with several genes (*HRAS*, *MACC1*, *MYC*, *SIRT1*, *TP53*, *ARNTL*, and *NR1D1*) in both SW480 *MACC1* OE and SW620 *MACC1* KO cells. These correlations were stronger in SW620 *MACC1* KO compared with the SW480 *MACC1* OE cells (Supplementary Figure S3B).

Interestingly, *MACC1* perturbations led to significant changes in the period, phase and amplitude of clock oscillation, as reported here for the first time (Figure 3C,D). While *MACC1* OE significantly increased the period of *ARNTL*-promoter activity in HCT116 (Figure 3D, ∆T*_MACC1_*_OE_ = 0.9 ± 0.1 h, mean ± SEM, *p* < 0.01, *n* = 3) and SW480 cells (Figure 4E, ∆T*_MACC1_*_OE_ = 0.8 ± 0.3 h, mean ± SEM, *p* < 0.05, *n* = 3), *MACC1* KO significantly decreased the period (Figure 3D, ∆T*_MACC1_*_KO_ = −0.8 ± 0.2 h, mean ± SEM, *p* < 0.01, *n* = 4). We did not detect reliable circadian oscillations in the SW620 cells, which was in line with previous reports for this cell line (Figure 4D) [25]. This opposing effect in the period of oscillations between *MACC1* OE and KO in HCT116 was also evident in the cell proliferation, migration and apoptosis data, with increased proliferation, migration and apoptosis upon overexpression of *MACC1* and an opposite effect upon *MACC1* KO (Figure 3E,F, *p* < 0.001, AUC compared with the respective control cell line). Altogether, these data point to a potential interaction between *MACC1* and the circadian clock network, which contributes to altered clock phenotype and cancer progression, possibly via core-clock components.

### 3.3. Cell Cycle Dynamics Are Altered in CRC upon KO of CC Genes

**Figure 5:**
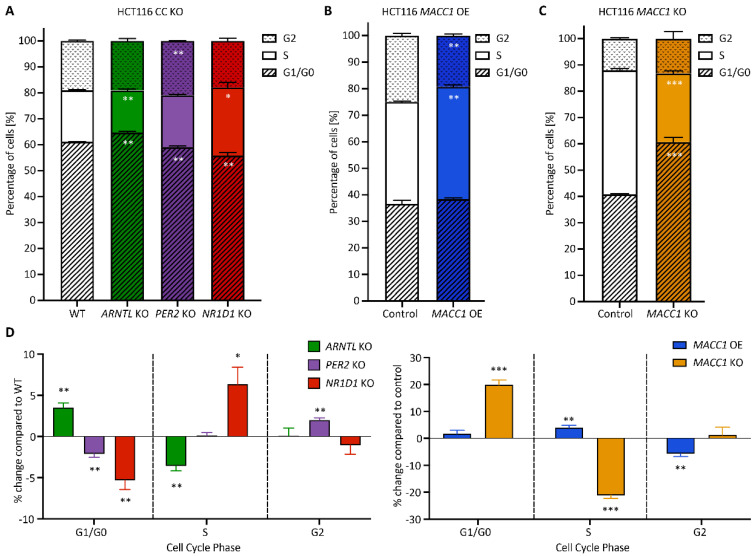
CC KO and *MACC1* manipulation alter cell cycle dynamics in HCT116. Cell cycle phase distribution in CC KO HCT116 cells (**A**) as well as (**B**) *MACC1* overexpressing and (**C**) *MACC1* KO HCT116 cells. (**D**) Normalized cell cycle phase distribution in CC KO and *MACC1* manipulated HCT116 cells compared with WT or control, respectively (*n* = 3, mean ± SEM, * *p* < 0.05, ** *p* < 0.01, *** *p* < 0.001; two-tailed unpaired *t*-test). Cells were synchronized and collected 24 h after synchronization.

Next, we explored the effects of CC KO, as well as *MACC1* manipulation on cell cycle dynamics. To do so, we analysed cell cycle phase distribution in synchronized cells and evaluated G1/G0, S or G2 phases compared with WT/Control cells (Figure 5). We observed significant changes in the cell cycle phase distribution in all CC KO cells compared with WT, as well as in *MACC1* manipulated cells compared with their respective controls. In particular, *ARNTL* KO cells displayed an increase in G1/G0 and a decrease in the S phase, whereas *PER2* KO and *NR1D1* KO led to a decrease in G1/G0 compared with WT (Figure 5A). In *MACC1* manipulated cells, we observed an increase in the number of cells in S and a decrease in the G2 phase upon *MACC1* OE (Figure 5B), whereas its KO led to a reduction of cells in S and an increased G1/G0 phase (Figure 5C). Comparing the changes in cell cycle between CC and *MACC1* manipulation, we found similarities between the KO of *ARNTL* and *MACC1*, both leading to more cells in G1/G0 and less in the S phase compared with the corresponding controls (Figure 5D). Overall, CC KO and *MACC1* manipulation affected cell cycle phase distributions in HCT116 cells with a KO specific role for CC genes.

### 3.4. Clock Alteration Affects MACC1 Rhythmic Protein Expression and Cell Invasion

**Figure 6:**
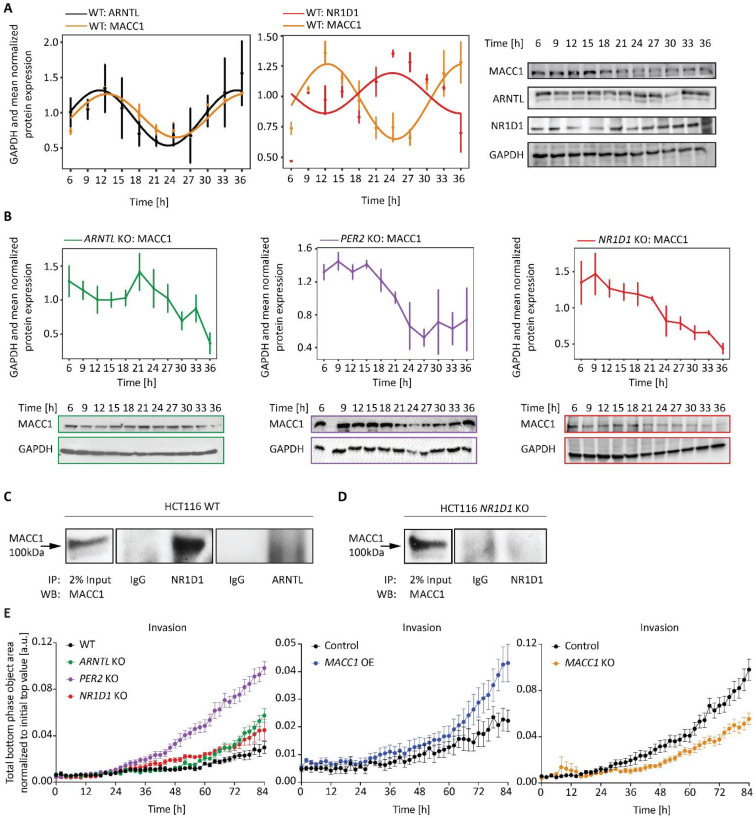
MACC1 protein oscillates similar to ARNTL and antiphase to NR1D1 in HCT116 cells. 30 h time-course protein expression via Western blotting in HCT116 WT (**A**) and core-clock KO cells (**B**). (**A**) ARNTL, NR1D1 and MACC1 expression in synchronized WT cells. (**B**) MACC1 expression in synchronized WT, *ARNTL* KO, *PER2* KO and *NR1D1* KO cells. GAPDH and mean normalized data is presented (*n* = 3, mean ± SEM). Rhythmicity analysis was performed using Cosinor in Discorhythm R package for a period of 24 h. A cosine curve was fitted for rhythmic data sets (*p* < 0.05), and data points were connected with closed lines for arrhythmic data points (*p* > 0.05). (**C**) Co-IP analysis of MACC1 binding to NR1D1 and not ARNTL in HCT116 WT and (**D**) *NR1D1* KO lysates. IP was performed for NR1D1 or ARNTL. Western blot was performed using MACC1 antibody. IgG: Isotype control. (**E**) Chemotaxis cell invasion analysis of HCT116 CC KO as well as *MACC1* OE and *MACC1* KO cells using IncuCyte S3 live-cell analysis. Cell invasion was quantified by measuring total phase object area on the bottom layer of the inner chamber normalized to the initial phase object area of the top layer within 84 h. Data presented as mean ± SEM (*n* = 8). Significance tested by comparing AUC with WT or the respective control cell line; two-tailed unpaired *t*-test. The original western blots were shown in File S1.

Based on the findings described above we sought to investigate whether MACC1 is clock-controlled and might display circadian expression. To test this, we generated time-course data of protein expression in synchronized HCT116 cells. Our data shows for the first time, that MACC1 protein has a rhythmic expression in HCT116 WT cells with a circadian period (Figure 6A, *n* = 3, mean ± SEM, *p* < 0.05), which closely follows ARNTL rhythms in these cells and oscillates antiphase to NR1D1 rhythms (Figure 6B). Interestingly, the KO of core-clock genes *ARNTL*, *PER2* or *NR1D1* all altered MACC1 rhythms and led to the disruption of 24 h rhythmic protein expression (Figure 6B).

To validate our hypothesis of a clock-MACC1 connection, we investigated the existence of possible protein–protein interactions between the core-clock and MACC1 via immunoprecipitation of NR1D1 and ARNTL from HCT116 WT lysate and tested for interactions with the MACC1 protein (Figure 6C,D). Indeed, our results indicated a MACC1-NR1D1 binding in WT (Figure 6C), which reinforced a clock-MACC1 connection at the protein level. This interaction was lost in *NR1D1* KO in HCT116 (Figure 6D).

We finally wondered whether CC or *MACC1* manipulation affects the invasive potential of CRC cells. For this, we measured chemotaxis cell invasion over time using live cell imaging in CC-KO and *MACC1* KO/OE HCT116 cells (Figure 6E, Supplementary Figure S4). Our results show that, indeed, all CC-KO led to a significant increase in the invasive capability of HCT116 cells (*n* = 8, mean ± SEM, *p* < 0.001), and the effect was more prominent in *PER2* KO and *ARNTL* KO cell lines. In *MACC1* manipulated cells, we observed a similar effect in *MACC1* OE cells with a significant higher invasive potential (*n* = 8, mean ± SEM, *p* < 0.01), which was opposite to the effect in the *MACC1* KO cell line (*n* = 8, mean ± SEM, *p* < 0.001). Hence, CC-KO cells with disrupted MACC1 rhythms showed increased invasiveness similar to *MACC1* OE cells.

Altogether, our results show evidence for the existence of a bi-directional interplay between MACC1 and the circadian clock, possibly through interactions with NR1D1, which might regulate CRC cell progression (e.g., proliferation and invasiveness).

## 4. Discussion

The circadian clock is known to affect several stages of cancer progression via interactions with cancer hallmarks, including cell growth, apoptosis, cell cycle and angiogenesis [40,41,42]. In CRC, studies have shown a role for the circadian clock in the context of cancer progression [13], including metastasis and metastatic-potential [22,43,44]. Low expression of *PER2* or *NR1D1* and upregulation of *CLOCK* are correlated with metastasis in CRC, as seen in patient (*PER2*, *CLOCK*) and in vivo (*NR1D1*, *CLOCK*) studies. Recently, it was shown that *ARNTL* affects CRC progression and metastasis by stimulating exosome secretion [45]. However, the mechanistic link between the clock and CRC progression is poorly investigated.

Here, we show that the circadian clock regulates elements related to cancer metastasis in CRC via CC genes. In particular, we report a strong differential expression of *MACC1*, a metastasis formation associated gene, upon CC KO. The increased expression of *MACC1* in *ARNTL* KO and *PER2* KO cells points to the intricate form of the core-clock network, as seen in our gene expression data for *ARNTL* and *PER2* in KO cell lines and highlights possible compensatory mechanisms, mainly within core-clock repressors, as previously reported [46].

MACC1 is a known driver for cancer metastasis and a prominent modulator of drug response in CRC [23,24,26]. It acts as a transcription factor regulating genes involved in EMT, such as c-MET (which can directly induce metastasis), impacts tumour cell migration and invasion, and induces metastasis in solid cancers [23]. However, to our knowledge, a connection between MACC1 and the circadian clock was not yet reported. Our data shows for the first time, that MACC1 is under circadian control and depicts oscillations in phase with ARNTL rhythms, which are lost after CC disruption (e.g., via the KO of *ARNTL*). We further speculate that the clock-MACC1 connection is mediated through NR1D1, which acts as an interacting partner for MACC1, as indicated by our immunoprecipitation assay.

In addition to disrupted MACC1 rhythms in CC KO cells, we observed an increased invasiveness potential in clock-disrupted cell lines by measuring chemotaxis cell invasion through a 3D biomatrix. In particular, *PER2* KO cells showed the highest increase in cell invasion, possibly also due to a significant increase in *MACC1* and a decrease in *ECAD* expression, leading to a more aggressive cancer phenotype by activating EMT markers. We observed a smaller increase in cell invasion in *ARNTL* KO cells, with a significant increase in both *MACC1* and *ECAD* expression.

Interestingly, our results also show that *MACC1* manipulation (KO or OE) affected CC genes expression, as well as the oscillation phenotype in CRC cells (as measured via bioluminescence recordings of *ARNTL*-promoter activity), reinforcing a clock-MACC1 connection. Significant changes in the periodicity of the circadian clock in CRC cells as well as altered CC gene expression upon manipulation of *MACC1* point to a bi-directional interplay between components of the circadian clock and MACC1. This highlights *MACC1* as a potential CCG, expanding the repertoire of CCGs involved in several hallmarks of cancer, including the cell cycle, proliferation and invasion, similar to MYC and RAS [8,10,47].

In a recent study, 258 CRC patients and 66 controls were analysed to evaluate the prognostic significance of CC proteins in CRC and to establish circadian clock biomarkers of CRC progression [48]. The study found that low expressions of ARNTL or PER2 were significantly associated with metastasis at the moment of disease diagnosis and suggested ARNTL and CRY1 as biomarkers of CRC patient survival and metastasis. These data corroborate our findings and highlight the role of CC genes in regulating cancer metastasis and invasion in CRC, possibly via interactions with *MACC1* on the level of gene expression and/or rhythmic oscillation.

The importance and potential benefits of circadian clock treatment, as well as timed therapy (i.e., chronotherapy) has been shown for several cancer types, including CRC [7,49,50], which reinforces the role of the circadian clock in tumour progression and genesis (reviewed in [51]). For example, pharmacological activation of REV-ERBs and RORs were reported to induce lethality in CRC [52,53] and a chrono-modulated FOLFOX treatment in metastatic CRC patients resulted in survival advantage over conventional treatment, especially in men [18]. In a recent systematic review of 18 clinical studies with 2547 cancer patients (e.g., colorectal, nasopharyngeal, endometrial and ovarian cancer) the authors concluded that chronomodulated chemotherapy resulted in reduced treatment toxicity while maintaining treatment efficacy in most cases (61% of the studies), compared with conventional therapy regimens [54]. Since MACC1 also acts as a therapeutic target restricting CRC progression and metastasis [24], a circadian MACC1 regulation is likely to affect the efficacy of treatment and would be expected to be circadian time-dependent. The results of the current study point to a strong interplay between cancer cell properties (e.g., proliferation and invasion) and the circadian clock via MACC1 in CRC. However, further analysis using primary cells from patient tumour samples, as well as subsequent studies in animal models are needed to verify these interactions in vivo, and their functional relevance in CRC.

## 5. Conclusions

Recent advancements in the field of chrono-oncology are beginning to unravel the connection between the circadian clock and cancer formation affecting treatment efficacy and patient outcome. Taken together, the results of the current study suggest the existence of a reciprocal interplay between MACC1 and the circadian clock, which plays an important role in the regulation of CRC cell proliferation and metastasis. Thus, these findings might be advantageous for the treatment of CRC, especially when targeting MACC1 and/or clock components in patients. Based on the promising results obtained in the current study, future investigations would be needed to validate our findings with in vivo models or with patient samples.

## Figures and Tables

**Table 1:** List of gRNA sequences and Cas9 plasmids used for core-clock (CC) KO in HCT116 cells. For each target gene, multiple gRNAs binding different genomic regions were used to increase KO efficiency.

Target Gene	gRNA Seq (without PAM)—(5′ –> 3′)	Cas9 Type
*ARNTL*	ACAGACAAAGATGACCCTCA	pSpCas9(BB)-2A-GFP
*ARNTL*	TTATCACACTACGGAGTCGA	pSpCas9(BB)-2A-GFP
*ARNTL*	CTGGACATTGCGTTGCATGT TAGATAAACTTACTGTGCTA	Cas9D10A-GFP (AIO-GFP)
*PER2*	GACCAACGAAAACTGCTCCA	pSpCas9(BB)-2A-GFP
*PER2*	GAACACAACCCATCTACAAG	pSpCas9(BB)-2A-GFP
*PER2*	CCCCGTGGAGCAGTTTTCGT GCAGTGACTGTGACGACAGT	Cas9D10A-GFP (AIO-GFP)
*NR1D1*	GTTGCGATTGATGCGGACGA	pSpCas9(BB)-2A-GFP
*NR1D1*	CGTAGGTGAAGATCTCTCGA	pSpCas9(BB)-2A-GFP

**Table 2:** List of potential predicted off-target regions using gRNAs targeting *ARNTL*, *PER2* or *NR1D1* with up to three mismatches within a protein-coding gene compared with the target region. PCR amplified products were Sanger-sequenced and compared with WT. * Primer also binds to other genomic regions (band sizes comparable to WT on gel electrophoresis).

Target Gene	gRNA Seq (No PAM)	#Mismatch	Region Type	Location	% Similarity Compared to WT
ARNTL	TTATCACACTACGGAGTCGA	3	intergenic		
ARNTL	ACAGACAAAGATGACCCTCA	3	exonic	16:89708928-89708950	100
PER2	GACCAACGAAAACTGCTCCA	3	intronic	6:157052704-157052726	100
PER2 *	GAACACAACCCATCTACAAG	3	intronic	2:115016664-115016686	-
PER2	GAACACAACCCATCTACAAG	3	intronic	7:4244707-4244729	100
PER2	GAACACAACCCATCTACAAG	3	intronic	3:161369567-161369589	100
NR1D1	CGTAGGTGAAGATCTCTCGA	3	intronic	12:99265850-99265872	100
NR1D1	GTTGCGATTGATGCGGACGA	3	intronic	17:20085554-20085576	100
NR1D1	GTTGCGATTGATGCGGACGA	3	exonic	8:144581166-144581188	100

**Table 3:** List and sequence of all primers designed in-house which were used for RT-qPCR analysis.

Target Gene	Forward Primer (5′–> 3′)	Reverse Primer (5′–> 3′)
*CD44*	ACACAAATGGCTGGTACGTCT	CCGTGGTGTGGTTGAAATGG
*CD133*	CCCCAGGAAATTTGAGG AAC	TCCAACAATCCATTCCCTGT
*ECAD*	ATTGCAAATTCCTGCCATTC	CTCTTCTCCGCCTCCTTCTT
*SIRT1*	AGGCCACGGATAGGTCCATA	GTGGAGGTATTGTTTCCGGC
*MACC1*	TTCTTTTGATTCCTCCGGTGA	ACTCTGATGGGCATGTGCTG

## Data Availability

The data presented in this study are available on request from the corresponding author.

## Supplementary Materials

The following supporting information can be downloaded at: https://www.mdpi.com/article/10.3390/cancers14143458/s1, Figure S1: CC-KO verification using CRISPR-Cas9 in HCT116 cells; Figure S2: Pearson correlation heatmaps between CC and EMT-related genes in HCT116 CC-KO cell lines; Figure S3: Pearson correlation heatmaps between CC and EMT-related genes in *MACC1* manipulated HCT116, SW480 and SW620 cell lines; Figure S4: CC KO and *MACC1* manipulation alters invasion capability in HCT116 cells, File S1: The original western blots.

## References

[1] Hanahan D., Weinberg R.A. (2011). Hallmarks of cancer: The next generation. Cell.

[2] Sulli G., Lam M.T.Y., Panda S. (2019). Interplay between Circadian Clock and Cancer: New Frontiers for Cancer Treatment. Trends Cancer.

[3] Zhang R., Lahens N.F., Ballance H.I., Hughes M.E., Hogenesch J.B. (2014). A circadian gene expression atlas in mammals: Implications for biology and medicine. Proc. Natl. Acad. Sci. USA.

[4] Mure L.S., Le H.D., Benegiamo G., Chang M.W., Rios L., Jillani N., Ngotho M., Kariuki T., Dkhissi-Benyahya O., Cooper H.M. (2018). Diurnal transcriptome atlas of a primate across major neural and peripheral tissues. Science.

[5] Takahashi J.S. (2017). Transcriptional architecture of the mammalian circadian clock. Nat. Rev. Genet..

[6] Patke A., Young M.W., Axelrod S. (2020). Molecular mechanisms and physiological importance of circadian rhythms. Nat. Rev. Mol. Cell Biol..

[7] Cederroth C.R., Albrecht U., Bass J., Brown S.A., Dyhrfjeld-Johnsen J., Gachon F., Green C.B., Hastings M.H., Helfrich-Forster C., Hogenesch J.B. (2019). Medicine in the Fourth Dimension. Cell Metab..

[8] Liu Z., Selby C.P., Yang Y., Lindsey-Boltz L.A., Cao X., Eynullazada K., Sancar A. (2020). Circadian regulation of c-MYC in mice. Proc. Natl. Acad. Sci. USA.

[9] Gotoh T., Kim J.K., Liu J., Vila-Caballer M., Stauffer P.E., Tyson J.J., Finkielstein C.V. (2016). Model-driven experimental approach reveals the complex regulatory distribution of p53 by the circadian factor Period 2. Proc. Natl. Acad. Sci. USA.

[10] Relogio A., Thomas P., Medina-Perez P., Reischl S., Bervoets S., Gloc E., Riemer P., Mang-Fatehi S., Maier B., Schafer R. (2014). Ras-mediated deregulation of the circadian clock in cancer. PLoS Genet..

[11] Tsuchiya Y., Minami I., Kadotani H., Todo T., Nishida E. (2013). Circadian clock-controlled diurnal oscillation of Ras/ERK signaling in mouse liver. Proc. Jpn. Acad. Ser. B Phys. Biol. Sci..

[12] Kinouchi K., Sassone-Corsi P. (2020). Metabolic rivalry: Circadian homeostasis and tumorigenesis. Nat. Rev. Cancer.

[13] Mazzoccoli G., Panza A., Valvano M.R., Palumbo O., Carella M., Pazienza V., Biscaglia G., Tavano F., Di Sebastiano P., Andriulli A. (2011). Clock gene expression levels and relationship with clinical and pathological features in colorectal cancer patients. Chronobiol. Int..

[14] Zeng Z.L., Luo H.Y., Yang J., Wu W.J., Chen D.L., Huang P., Xu R.H. (2014). Overexpression of the circadian clock gene Bmal1 increases sensitivity to oxaliplatin in colorectal cancer. Clin. Cancer Res..

[15] Gery S., Komatsu N., Baldjyan L., Yu A., Koo D., Koeffler H.P. (2006). The circadian gene per1 plays an important role in cell growth and DNA damage control in human cancer cells. Mol. Cell.

[16] Huber A.L., Papp S.J., Chan A.B., Henriksson E., Jordan S.D., Kriebs A., Nguyen M., Wallace M., Li Z., Metallo C.M. (2016). CRY2 and FBXL3 Cooperatively Degrade c-MYC. Mol. Cell.

[17] Yu H., Meng X., Wu J., Pan C., Ying X., Zhou Y., Liu R., Huang W. (2013). Cryptochrome 1 overexpression correlates with tumor progression and poor prognosis in patients with colorectal cancer. PLoS ONE.

[18] Giacchetti S., Bjarnason G., Garufi C., Genet D., Iacobelli S., Tampellini M., Smaaland R., Focan C., Coudert B., Humblet Y. (2006). Phase III trial comparing 4-day chronomodulated therapy versus 2-day conventional delivery of fluorouracil, leucovorin, and oxaliplatin as first-line chemotherapy of metastatic colorectal cancer: The European Organisation for Research and Treatment of Cancer Chronotherapy Group. J. Clin. Oncol..

[19] Innominato P.F., Karaboue A., Focan C., Chollet P., Giacchetti S., Bouchahda M., Ulusakarya A., Torsello A., Adam R., Levi F.A. (2020). Efficacy and safety of chronomodulated irinotecan, oxaliplatin, 5-fluorouracil and leucovorin combination as first- or second-line treatment against metastatic colorectal cancer: Results from the International EORTC 05011 Trial. Int. J. Cancer.

[20] Innominato P., Komarzynski S., Karaboue A., Ulusakarya A., Bouchahda M., Haydar M., Bossevot-Desmaris R., Mocquery M., Plessis V., Levi F. (2018). Home-Based e-Health Platform for Multidimensional Telemonitoring of Symptoms, Body Weight, Sleep, and Circadian Activity: Relevance for Chronomodulated Administration of Irinotecan, Fluorouracil-Leucovorin, and Oxaliplatin at Home-Results From a Pilot Study. JCO Clin. Cancer Inform..

[21] Innominato P.F., Komarzynski S., Palesh O.G., Dallmann R., Bjarnason G.A., Giacchetti S., Ulusakarya A., Bouchahda M., Haydar M., Ballesta A. (2018). Circadian rest-activity rhythm as an objective biomarker of patient-reported outcomes in patients with advanced cancer. Cancer Med..

[22] Basti A., Fior R., Yalin M., Povoa V., Astaburuaga R., Li Y., Naderi J., Godinho Ferreira M., Relogio A. (2020). The Core-Clock Gene NR1D1 Impacts Cell Motility In Vitro and Invasiveness in A Zebrafish Xenograft Colon Cancer Model. Cancers.

[23] Stein U., Walther W., Arlt F., Schwabe H., Smith J., Fichtner I., Birchmeier W., Schlag P.M. (2009). MACC1, a newly identified key regulator of HGF-MET signaling, predicts colon cancer metastasis. Nat. Med..

[24] Radhakrishnan H., Walther W., Zincke F., Kobelt D., Imbastari F., Erdem M., Kortum B., Dahlmann M., Stein U. (2018). MACC1-the first decade of a key metastasis molecule from gene discovery to clinical translation. Cancer Metastasis Rev..

[25] Fuhr L., El-Athman R., Scrima R., Cela O., Carbone A., Knoop H., Li Y., Hoffmann K., Laukkanen M.O., Corcione F. (2018). The Circadian Clock Regulates Metabolic Phenotype Rewiring Via HKDC1 and Modulates Tumor Progression and Drug Response in Colorectal Cancer. EBioMedicine.

[26] Juneja M., Kobelt D., Walther W., Voss C., Smith J., Specker E., Neuenschwander M., Gohlke B.O., Dahlmann M., Radetzki S. (2017). Statin and rottlerin small-molecule inhibitors restrict colon cancer progression and metastasis via MACC1. PLoS Biol..

[27] Dahlmann M., Werner R., Kortum B., Kobelt D., Walther W., Stein U. (2020). Restoring Treatment Response in Colorectal Cancer Cells by Targeting MACC1-Dependent ABCB1 Expression in Combination Therapy. Front. Oncol..

[28] Pliatsika V., Rigoutsos I. (2015). “Off-Spotter”: Very fast and exhaustive enumeration of genomic lookalikes for designing CRISPR/Cas guide RNAs. Biol. Direct..

[29] Hodgkins A., Farne A., Perera S., Grego T., Parry-Smith D.J., Skarnes W.C., Iyer V. (2015). WGE: A CRISPR database for genome engineering. Bioinformatics.

[30] Maier B., Lorenzen S., Finger A.M., Herzel H., Kramer A. (2021). Searching Novel Clock Genes Using RNAi-Based Screening. Methods Mol. Biol..

[31] Schneider C.A., Rasband W.S., Eliceiri K.W. (2012). NIH Image to ImageJ: 25 years of image analysis. Nat. Methods.

[32] Carlucci M., Krisciunas A., Li H., Gibas P., Koncevicius K., Petronis A., Oh G. (2021). DiscoRhythm: An easy-to-use web application and R package for discovering rhythmicity. Bioinformatics.

[33] McKenzie A.T., Katsyv I., Song W.M., Wang M., Zhang B. (2016). DGCA: A comprehensive R package for Differential Gene Correlation Analysis. BMC Syst. Biol..

[34] Bunger M.K., Wilsbacher L.D., Moran S.M., Clendenin C., Radcliffe L.A., Hogenesch J.B., Simon M.C., Takahashi J.S., Bradfield C.A. (2000). Mop3 is an essential component of the master circadian pacemaker in mammals. Cell.

[35] Wu Y., Tian T., Wu Y., Yang Y., Zhang Y., Qin X. (2021). Systematic Studies of the Circadian Clock Genes Impact on Temperature Compensation and Cell Proliferation Using CRISPR Tools. Biology.

[36] El-Athman R., Fuhr L., Relogio A. (2018). A Systems-Level Analysis Reveals Circadian Regulation of Splicing in Colorectal Cancer. EBioMedicine.

[37] Mazzoccoli G., Colangelo T., Panza A., Rubino R., De Cata A., Tiberio C., Valvano M.R., Pazienza V., Merla G., Augello B. (2016). Deregulated expression of cryptochrome genes in human colorectal cancer. Mol. Cancer.

[38] Serchov T., Jilg A., Wolf C.T., Radtke I., Stehle J.H., Heumann R. (2016). Ras Activity Oscillates in the Mouse Suprachiasmatic Nucleus and Modulates Circadian Clock Dynamics. Mol. Neurobiol..

[39] Chang H.C., Guarente L. (2013). SIRT1 mediates central circadian control in the SCN by a mechanism that decays with aging. Cell.

[40] Shafi A.A., Knudsen K.E. (2019). Cancer and the Circadian Clock. Cancer Res..

[41] Lee Y. (2021). Roles of circadian clocks in cancer pathogenesis and treatment. Exp. Mol. Med..

[42] Sancar A., Lindsey-Boltz L.A., Gaddameedhi S., Selby C.P., Ye R., Chiou Y.Y., Kemp M.G., Hu J., Lee J.H., Ozturk N. (2015). Circadian clock, cancer, and chemotherapy. Biochemistry.

[43] Oshima T., Takenoshita S., Akaike M., Kunisaki C., Fujii S., Nozaki A., Numata K., Shiozawa M., Rino Y., Tanaka K. (2011). Expression of circadian genes correlates with liver metastasis and outcomes in colorectal cancer. Oncol. Rep..

[44] Wang Y., Sun N., Lu C., Bei Y., Qian R., Hua L. (2017). Upregulation of circadian gene ‘hClock’ contribution to metastasis of colorectal cancer. Int. J. Oncol..

[45] Dong P., Wang Y., Liu Y., Zhu C., Lin J., Qian R., Hua L., Lu C. (2022). BMAL1 induces colorectal cancer metastasis by stimulating exosome secretion. Mol. Biol. Rep..

[46] Baggs J.E., Price T.S., DiTacchio L., Panda S., Fitzgerald G.A., Hogenesch J.B. (2009). Network features of the mammalian circadian clock. PLoS Biol..

[47] Altman B.J., Hsieh A.L., Sengupta A., Krishnanaiah S.Y., Stine Z.E., Walton Z.E., Gouw A.M., Venkataraman A., Li B., Goraksha-Hicks P. (2015). MYC Disrupts the Circadian Clock and Metabolism in Cancer Cells. Cell Metab..

[48] Aroca-Siendones M.I., Moreno-SanJuan S., Puentes-Pardo J.D., Verbeni M., Arnedo J., Escudero-Feliu J., Garcia-Costela M., Garcia-Robles A., Carazo A., Leon J. (2021). Core Circadian Clock Proteins as Biomarkers of Progression in Colorectal Cancer. Biomedicines.

[49] Sulli G., Manoogian E.N.C., Taub P.R., Panda S. (2018). Training the Circadian Clock, Clocking the Drugs, and Drugging the Clock to Prevent, Manage, and Treat Chronic Diseases. Trends Pharmacol. Sci..

[50] Battaglin F., Chan P., Pan Y., Soni S., Qu M., Spiller E.R., Castanon S., Roussos Torres E.T., Mumenthaler S.M., Kay S.A. (2021). Clocking cancer: The circadian clock as a target in cancer therapy. Oncogene.

[51] Sancar A., Van Gelder R.N. (2021). Clocks, cancer, and chronochemotherapy. Science.

[52] Sulli G., Rommel A., Wang X., Kolar M.J., Puca F., Saghatelian A., Plikus M.V., Verma I.M., Panda S. (2018). Pharmacological activation of REV-ERBs is lethal in cancer and oncogene-induced senescence. Nature.

[53] Ashrafizadeh M., Zarrabi A., Saberifar S., Hashemi F., Hushmandi K., Hashemi F., Moghadam E.R., Mohammadinejad R., Najafi M., Garg M. (2020). Nobiletin in Cancer Therapy: How This Plant Derived-Natural Compound Targets Various Oncogene and Onco-Suppressor Pathways. Biomedicines.

[54] Printezi M.I., Kilgallen A.B., Bond M.J.G., Stibler U., Putker M., Teske A.J., Cramer M.J., Punt C.J.A., Sluijter J.P.G., Huitema A.D.R. (2022). Toxicity and efficacy of chronomodulated chemotherapy: A systematic review. Lancet Oncol..

[55] Chiang T.W., Le Sage C., Larrieu D., Demir M., Jackson S.P. (2016). CRISPR-Cas9(D10A) nickase-based genotypic and phenotypic screening to enhance genome editing. Sci. Rep..

