# Supplementary material for: Core-Clock Genes Regulate Proliferation and Invasion via a Reciprocal Interplay with MACC1 in Colorectal Cancer Cells
Source: Cancers (Basel). 2022 Jul 16;14(14):3458. doi: 10.3390/cancers14143458 (PMC9319284; doi:10.3390/cancers14143458)
Supplement: Supplementary file 1 [file cancers-14-03458-s001.zip › File S1.pdf]

SUPPLEMENTARY MATERIALS

Raw western blot images:

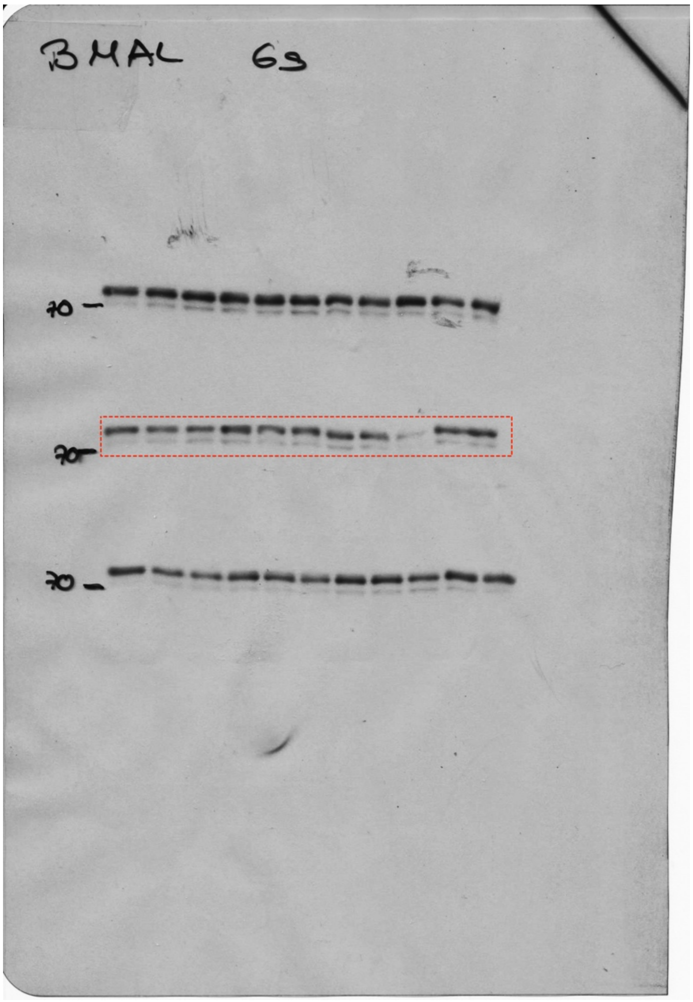

Figure 6A: WT-ARNTL

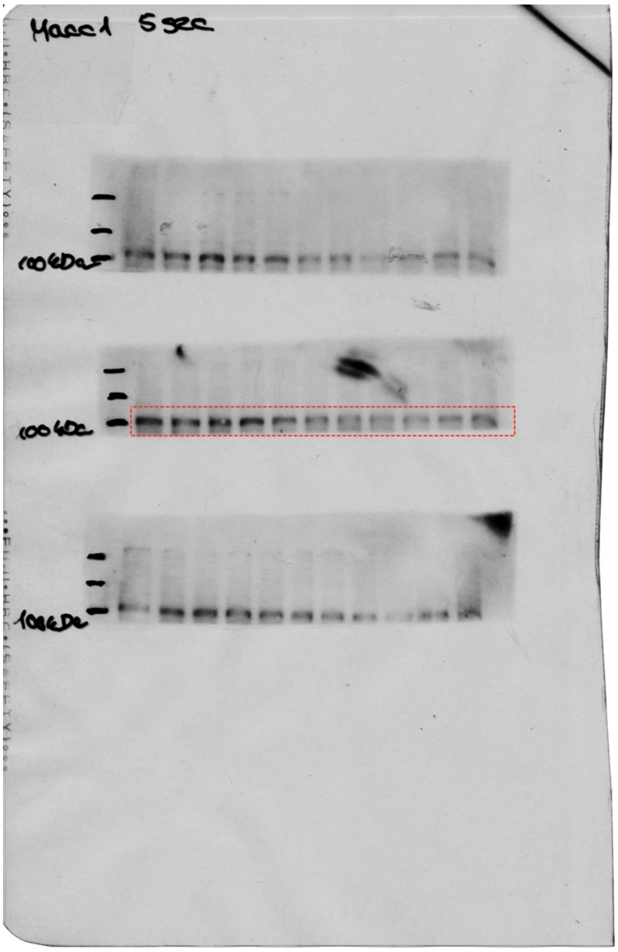

Figure 6A: WT-MACC1

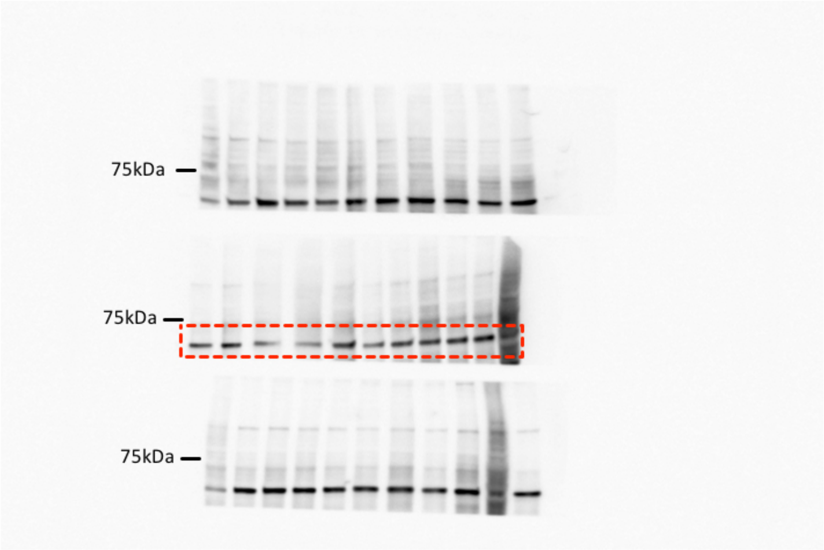

Figure 6A: WT-NR1D1

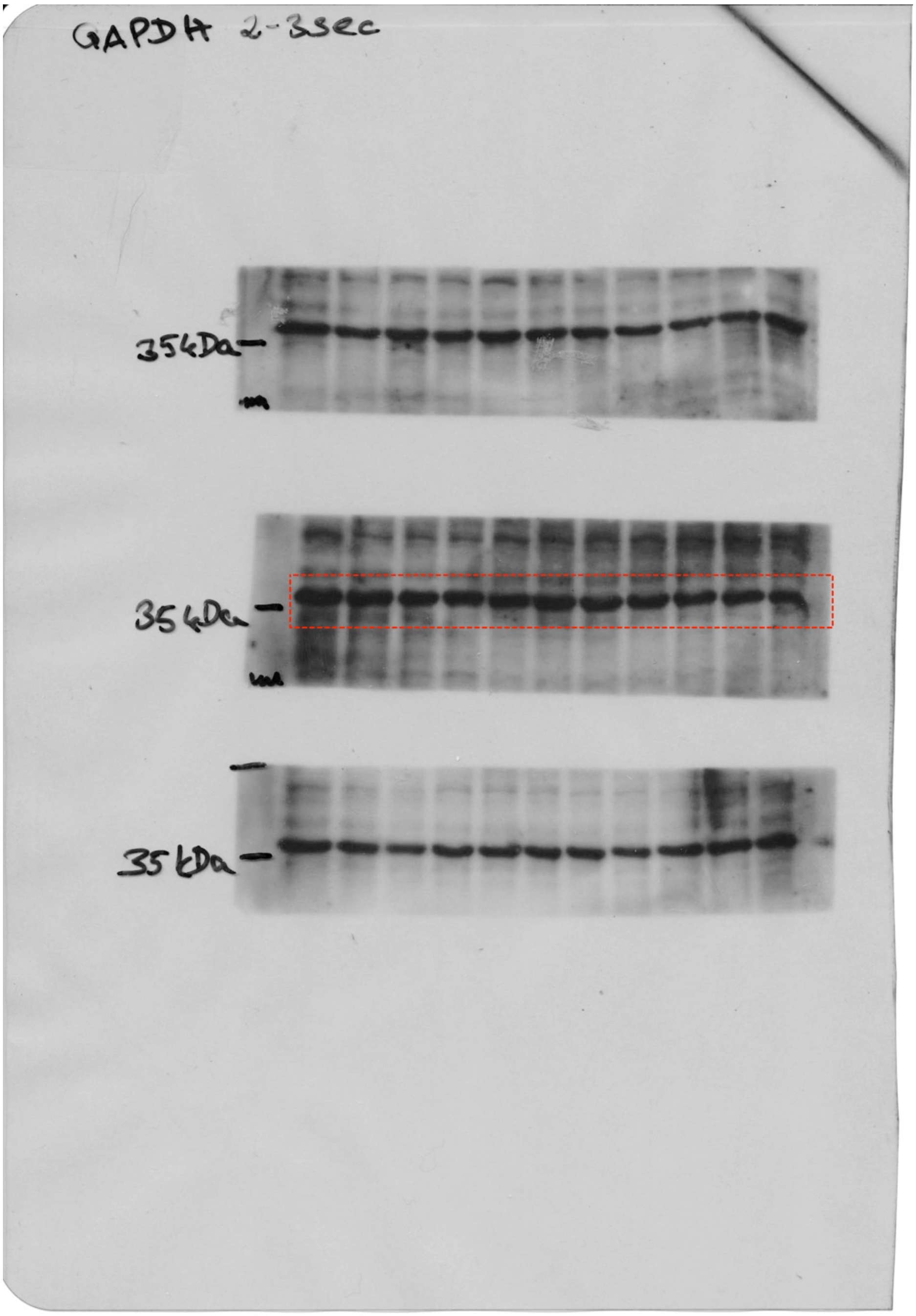

Figure 6A: WT-GAPDH

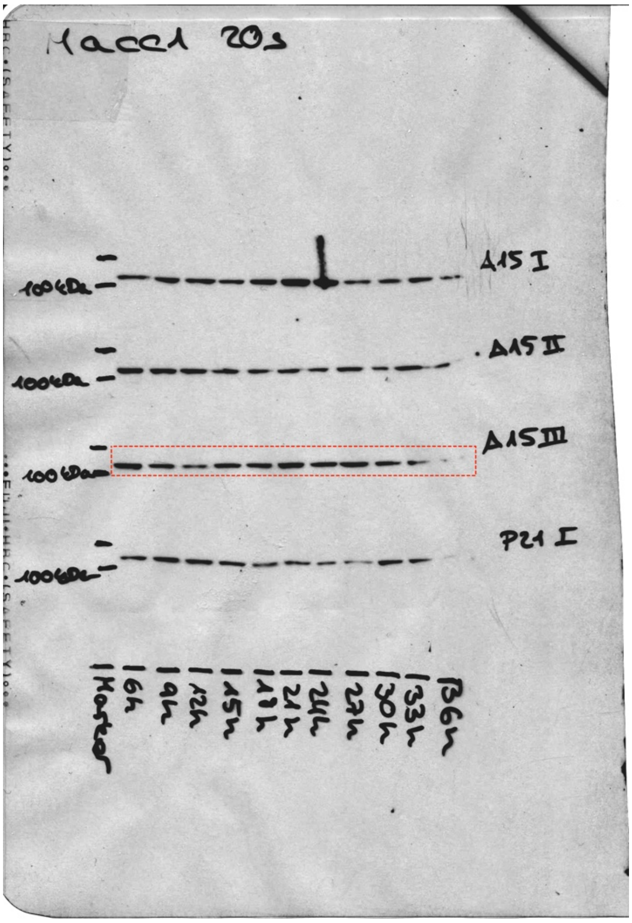

Figure 6B: ARNTL KO-MACC1

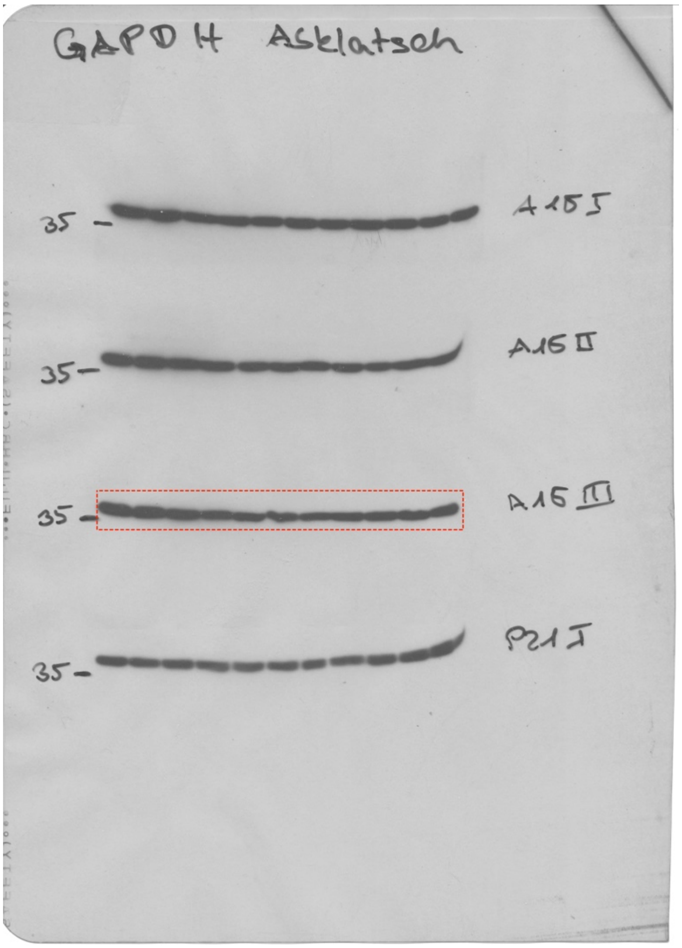

Figure 6B: ARNTL KO-GAPDH

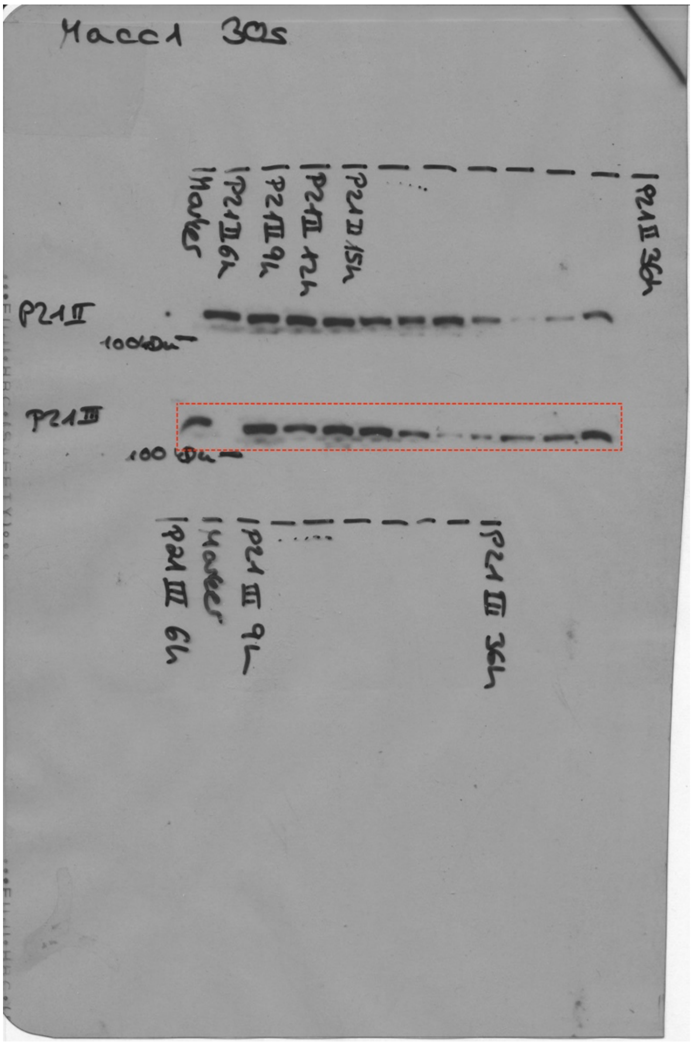

Figure 6B: PER2 KO-MACC1

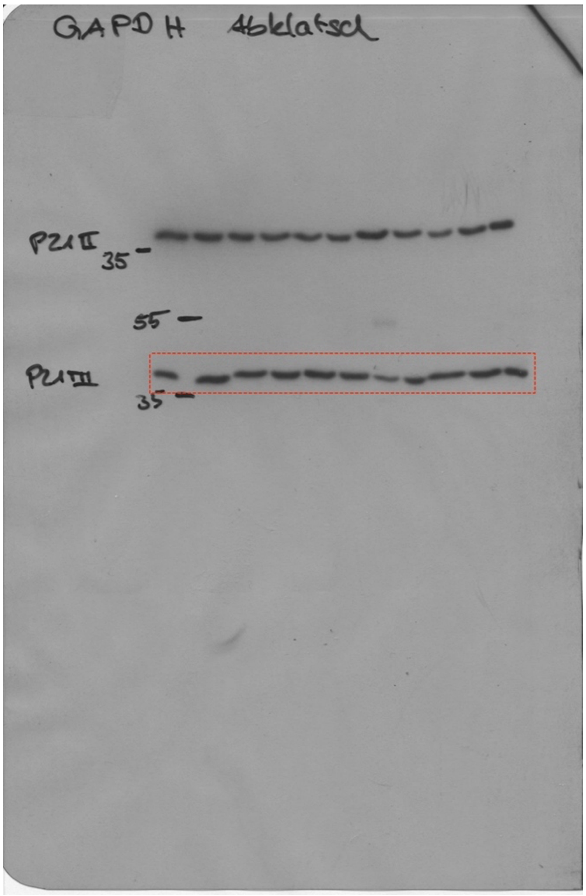

Figure 6B: PER2 KO-GAPDH

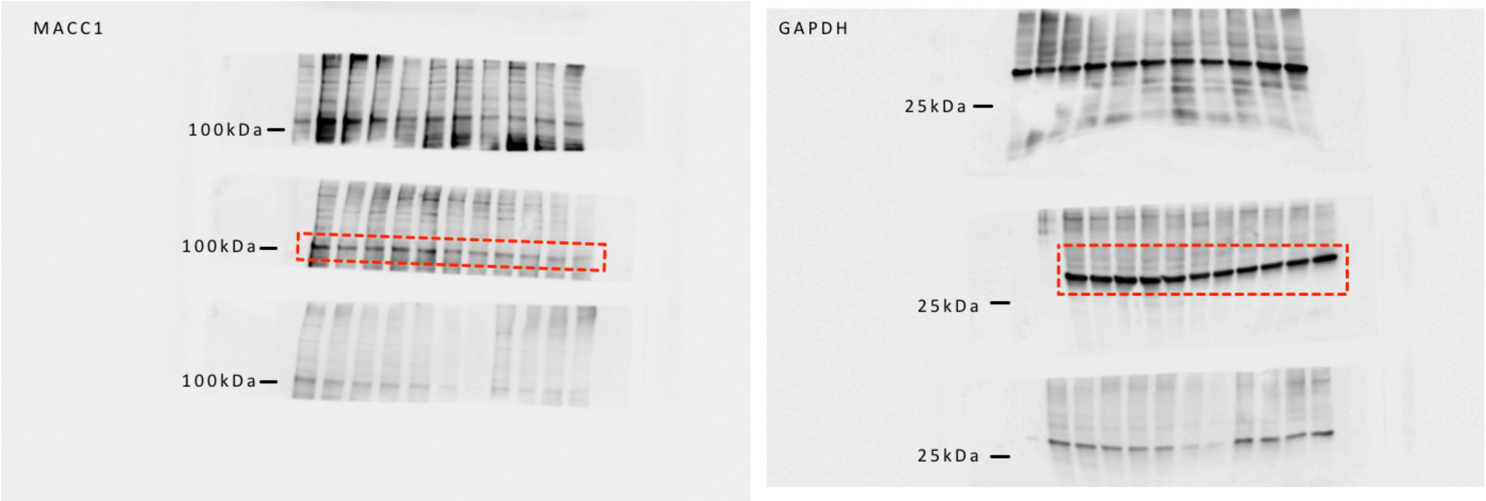

Figure 6B: NR1D1 KO-MACC1 & GAPDH

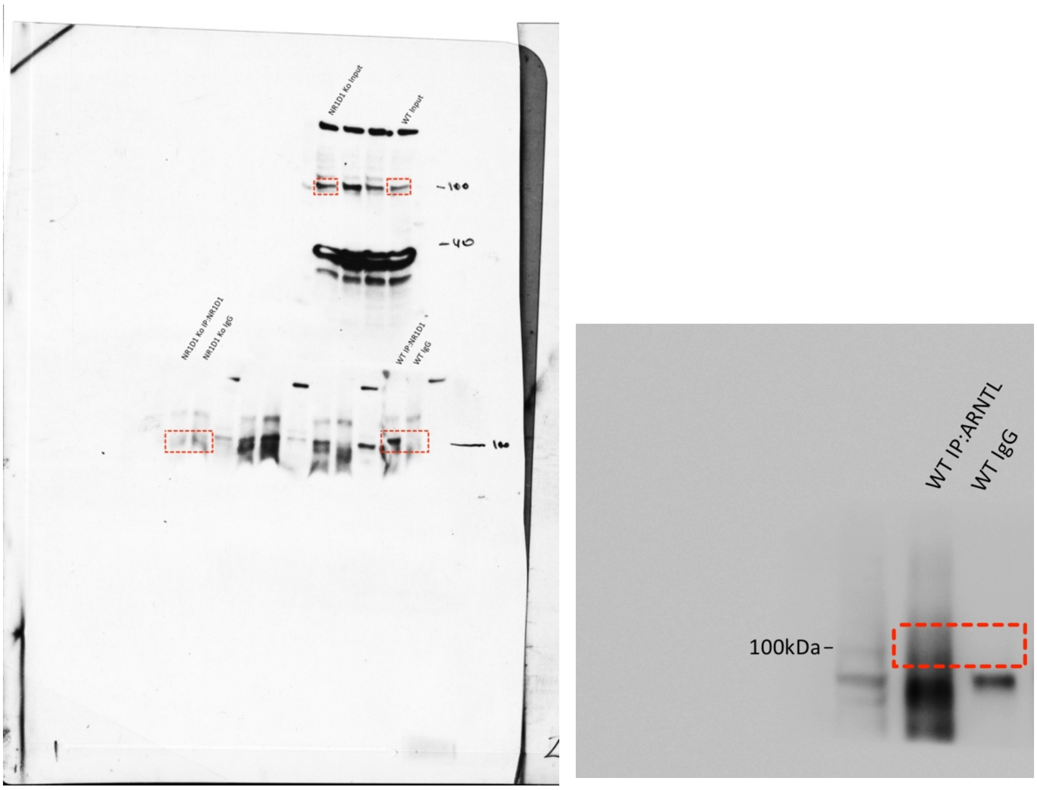

Figure 6C-D: Co-IP: IP with NR1D1 or ARNTL, WB with MACC1 in HCT116 WT and NR1D1 KO

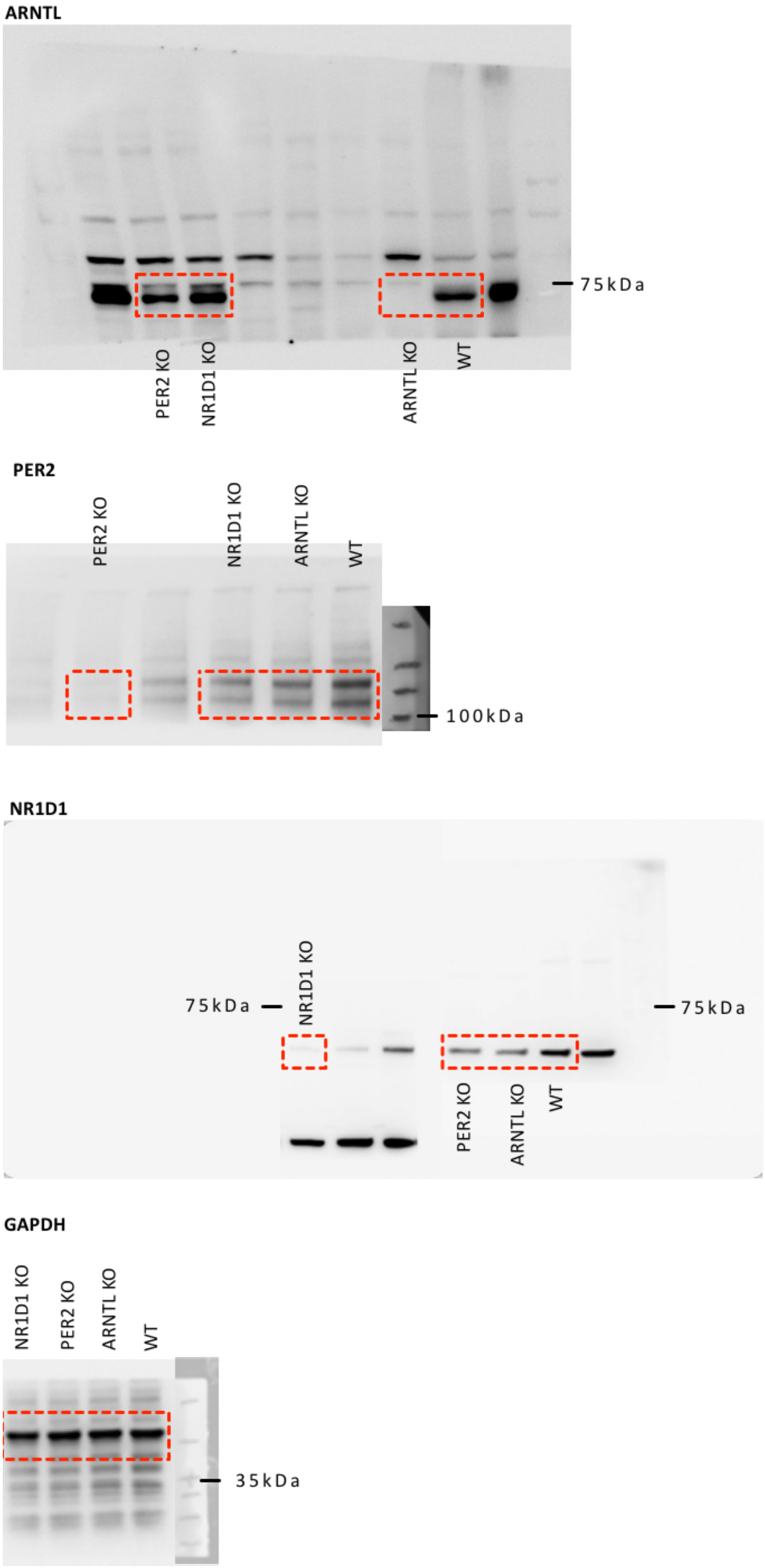

Figure S1-E: WT, ARNTL KO, PER2 KO and NR1D1 KO.
