# Supplementary material for: Core-Clock Genes Regulate Proliferation and Invasion via a Reciprocal Interplay with MACC1 in Colorectal Cancer Cells
Source: Cancers (Basel). 2022 Jul 16;14(14):3458. doi: 10.3390/cancers14143458 (PMC9319284; doi:10.3390/cancers14143458)
Supplement: Supplementary file 1 [file cancers-14-03458-s001.zip › Supplementary Figures.pdf]

SUPPLEMENTARY MATERIALS

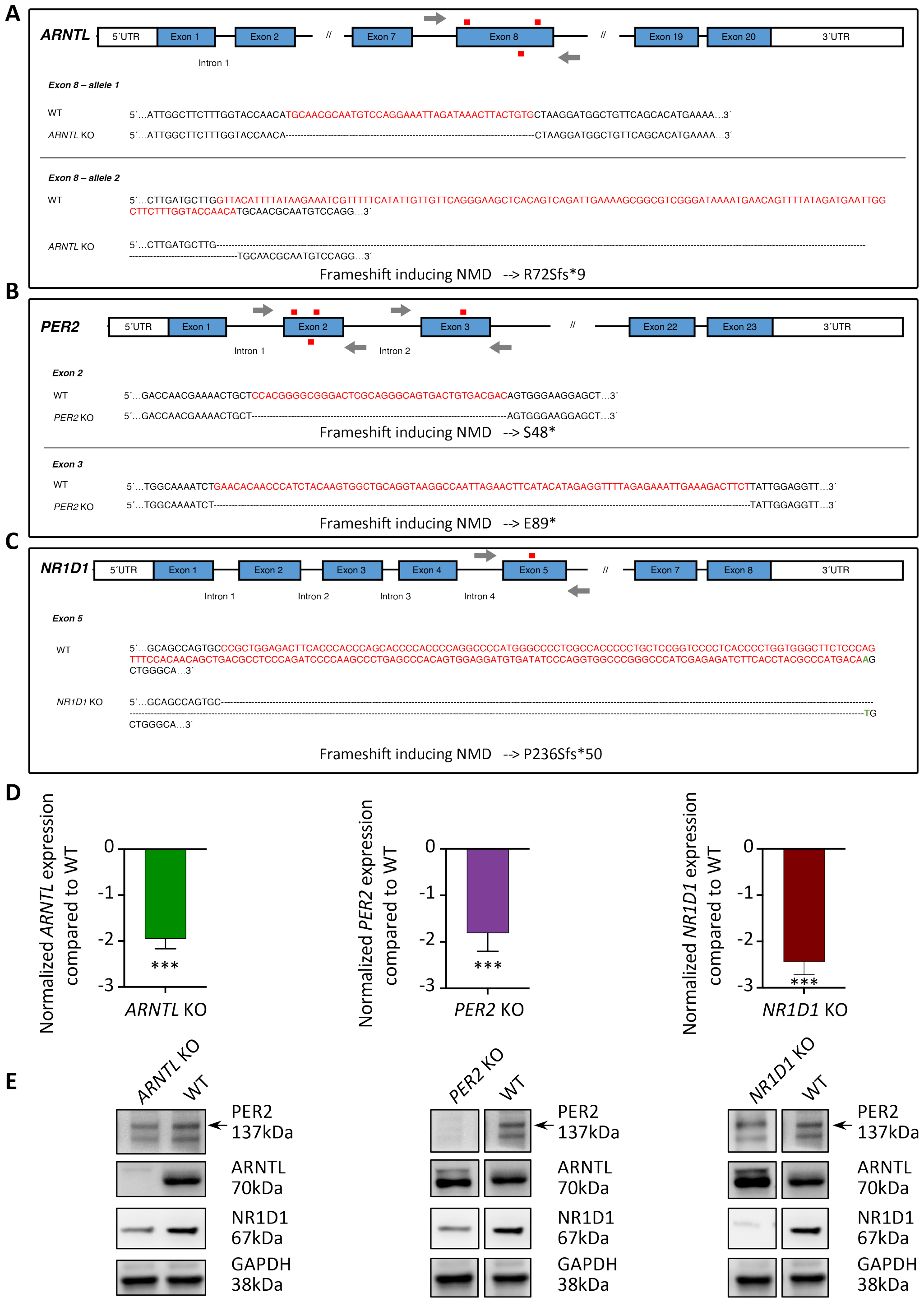

Figure S1: CC-KO verification using CRISPR-Cas9 in HCT116 cells. Schematic representation of the position of guide RNAs (gRNA) for the CRISPR-Cas9 knockout (KO) assay in HCT116 cells and the resulting sequence alteration in selected mutants. (A-C) For each target gene (*ARNTL*,

PER2 or *NR1D1*) guide RNAs were designed targeting exons close to the 5´region of the gene (red squares). For selected candidate knockout mutants, the genomic region (DNA) around the gRNA binding site (indicated by grey arrows) was sequenced and compared to the WT gene sequence. Red-marked bases indicate deletion in the KO mutant. Green-marked base indicate base substitution between WT and KO mutant. NMD = nonsense-mediated decay; UTR = Untranslated region. Knockout verification using mRNA (D) as well protein (E) expression of core-clock genes in selected mutant clones compared to the WT. n = 3, *** $p < 0.001$.

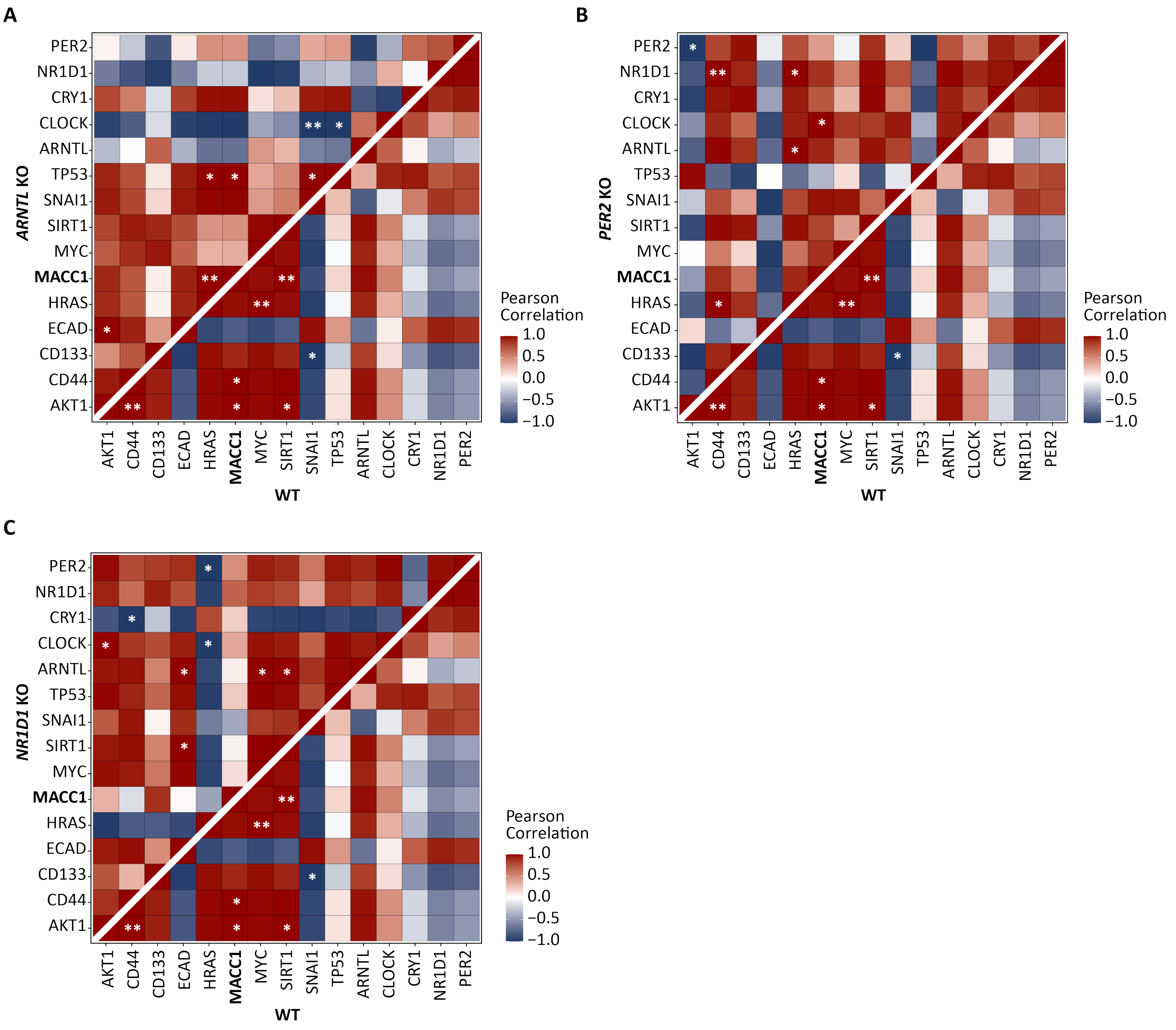

Figure S2: Pearson correlation heatmaps between CC and EMT-related genes in HCT116 CC-KO cell lines. Heatmaps of Pearson correlation between each pair of core-clock and EMT-related genes for HCT116 WT versus (A) HCT116 *ARNTL* KO, (B) *PER2* KO and (C) *NR1D1* KO cells. * $p < 0.05$, ** $p < 0.01$, *** $p < 0.001$; two-tailed unpaired t-test.

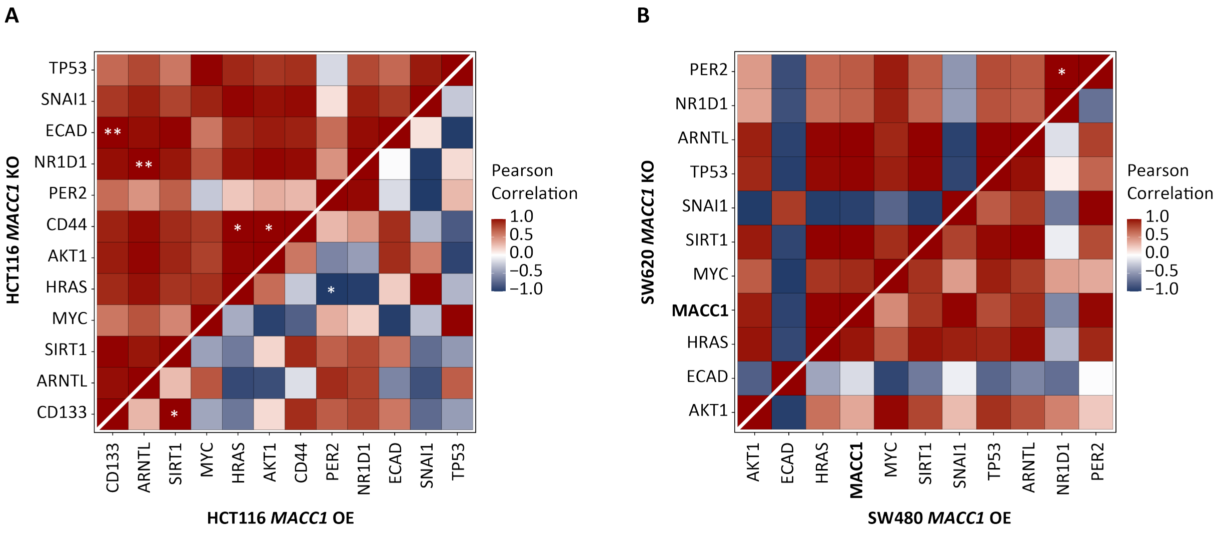

Figure S3: Pearson correlation heatmaps between CC and EMT-related genes in *MACC1* manipulated HCT116, SW480 and SW620 cell lines. Heatmaps of Pearson correlation between each pair of core-clock and EMT-related genes for (A) HCT116 *MACC1* KO versus HCT116 *MACC1* OE and (B) SW620 *MACC1* KO versus SW480 *MACC1* OE cells. * $p < 0.05$, ** $p < 0.01$, *** $p < 0.001$; two-tailed unpaired t-test.

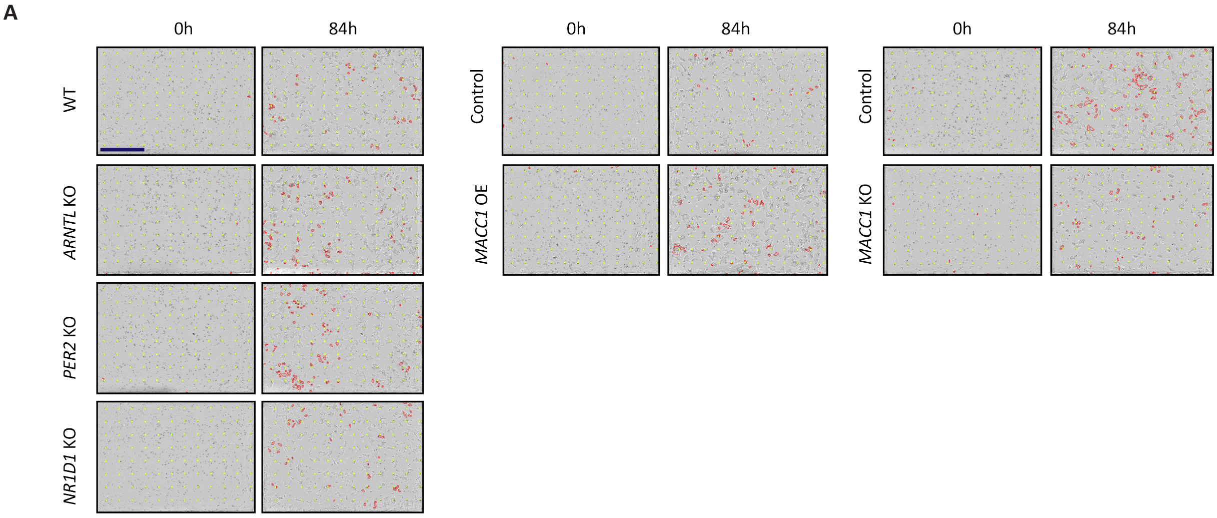

Figure S4: CC KO and *MACC1* manipulation alters invasion capability in HCT116 cells. (A) Representative images of the chemotaxis cell invasion assay from the bottom view. Red mask shows cell area on the bottom layer of the inner chamber. Green dots highlights the pores of the inner chamber. Scale bar = 700 µm
